# Distinct patterns of endothelial response to endotoxin in aged mice as compared to young mice

**DOI:** 10.1007/s11357-025-01838-9

**Published:** 2025-11-26

**Authors:** Joanna Suraj-Prażmowska, Magdalena Sternak, Anna Kurpińska, Anna Bar, Izabela Czyżyńska-Cichoń, Kelly Ascencao, Ewa Niedzielska-Andres, Elżbieta Buczek, Łukasz Mateuszuk, Agnieszka Karaś, Maria Walczak, Csaba Szabo, Stefan Chlopicki

**Affiliations:** 1 Jagiellonian University, Jagiellonian Centre for Experimental Therapeutics (JCET), Krakow, Poland; 2 Jagiellonian University Medical College, Department of Pharmacology, Krakow, Poland; 3 Jagiellonian University, Jagiellonian University Medical College, Faculty of Pharmacy, Chair and Department of Toxicology, Krakow, Poland; 4 University of Fribourg, Faculty of Science and Medicine, Department of Oncology, Microbiology and Immunology, Section of Pharmacology, Fribourg, Switzerland

**Keywords:** Endothelium, Endotoxemia, Vascular aging, Biomarkers, Inflammation, Hemostasis, Targeted proteomics

## Abstract

Aging exacerbates organ injury in endotoxemia, but it is not clear whether endotoxemia is associated with a specific, age-related profile of the endothelial response. Therefore, the aim of the study was to assess the pattern of endothelial response to lipopolysaccharide (LPS) in aged mice (18-month-old) as compared to young mice (3-month-old). Our analysis was based on functional endothelial responses measured in vivo by magnetic resonance imaging (MRI) and on a comprehensive panel of biomarkers of endothelial dysfunction measured by micro-flow liquid chromatography tandem mass spectrometry (microLC-MS/MS). In aged mice, the systemic inflammatory response (serum amyloid A, IL-1β, IL-2, eotaxin), kidney injury (urea), liver injury (ALT), and endothelial dysfunction induced by a relatively low dose of LPS (3 mg/kg) were all more pronounced as compared with young mice. Interestingly, in aged mice, LPS induced a different pattern of endothelial response compared to young mice, as evidenced by glycocalyx injury biomarkers (SDC-1, ESM-1), the endothelial permeability biomarkers (Angpt-2, sTie-2) and various hemostasis-related factors (sTM, TAFI, THBS-1). In contrast, biomarkers of endothelial inflammation (sVCAM-1, sICAM-1, sE-selectin, sP-selectin) and classical hemostasis biomarkers (PAI-1, t-PA, von Willebrand factor) displayed comparable responses to LPS in aged and young mice. In conclusion, aging does not indiscriminately potentiate LPS-induced inflammatory mediator generation in the current model of endotoxemia induced by a relatively low dose of LPS, but selectively alters the endothelial response in terms of glycocalyx injury, endothelial permeability, and hemostasis.

**Graphical Abstract:**

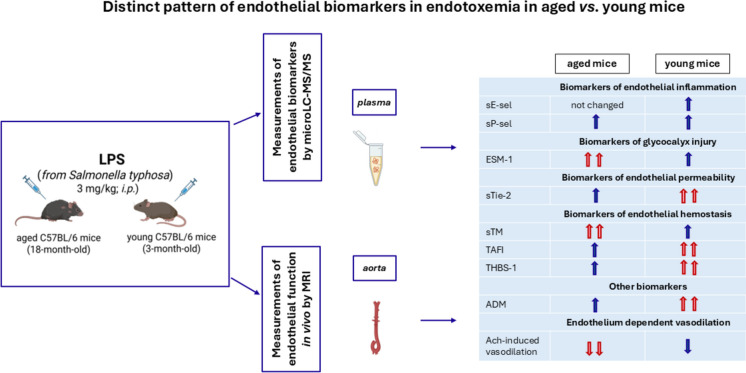

**Supplementary Information:**

The online version contains supplementary material available at 10.1007/s11357-025-01838-9.

## Introduction

Endothelial activation and subsequent dysfunction play key roles in the pathogenesis of sepsis. Endothelial dysfunction–defined as impaired nitric oxide (NO)-dependent function-has a prognostic value in sepsis and is associated with many pathophysiological changes, including inflammatory response, oxidative stress, and disseminated intravascular coagulation (DIC) [1–3]. In sepsis, endothelial dysfunction contributes to impaired microcirculation perfusion and organ dysfunction [4].

It is well known that aging is accompanied by a reduced tolerance to various stressors and insults, which may contribute to increased susceptibility of aged individuals to critical illnesses including sepsis and septic shock [5]. Importantly, sepsis in aged individuals is associated with higher mortality [6, 7] and this is also recapitulated in preclinical models: endotoxemia in aged mice results in higher mortality rate than in young mice [8].

The increased severity of sepsis in older patients may be related to various comorbidities or to a distinct pathogenesis of progression of sepsis in older individuals [9]. The unique pathophysiology of sepsis observed in older patients is generally attributed to coagulation abnormalities, dysfunctional immune system, inflammaging, and exacerbated cytokine production [5]. Several of experimental studies in animals investigated various mechanisms that could contribute to increased susceptibility of organ injury in aging. For example, eNOS-deficient and aged mice were found to exhibit a higher degree of sepsis-associated multiple organ dysfunction syndrome (MODS), infiltration of tissues with mononuclear cells, and oxidative stress [10, 11], which may be relevant to age-dependent deterioration of NO-dependent function [12–14]. Similarly, vascular aging was associated with a reduced antioxidant defense, as evidenced by the downregulation of various antioxidant enzymes (e.g., SOD), as well as reduced levels of antioxidant factors (e.g., glutathione) [15–17]. A key component of this attenuated antioxidant response in aging was related to the age dependence in the activation of nuclear factor erythroid 2-related factor 2 (Nrf2), a key redox regulatory factor. The induction of this factor was prominent in young organisms, but it was suppressed in aging [18–22].

Based on the accumulated knowledge on pathophysiology of aging, various prognostic sepsis biomarkers have been identified, which may have utility in predicting sepsis severity and organ damage as well as mortality in patients. These markers include granulocyte colony-stimulating factor (G-CSF) [23, 24], angiotensin II (Ang-2), interleukin-1 receptor antagonist (IL-1ra) and pentraxin 3 (PTX-3) [25], insulin-like growth factor-1 (IGF‑1) [26], sirtuin 3 (Sirt3) [27], thrombomodulin (TM) [28–30], soluble form of thrombomodulin (sTM) and syndecan-1 (SDC-1) [31], angiopoietin 2 (Angpt-2) [32], and others [33, 34]. However, the available information is relatively limited regarding the age-dependent regulation of various pathomechanisms in sepsis. Previously, age-dependent changes in pathways related to protein C (PC), thrombomodulin-dependent mechanisms [8], glucose/IGF-1 signaling pathway [35], and the profile of systemic inflammatory response were suggested [36].

Endothelial dysfunction has a prognostic value in sepsis [37]. However, it is still not clear whether the age-dependent increase in vulnerability for LPS-induced organ injury is associated with specific-age dependent profile of the endothelial response to LPS. Therefore, the aim of the present study was to assess the endothelial response to LPS in aged mice as compared with young mice using functional (MRI-based) and biochemical (microLC-MS/MS-based) endothelial profiling. The intensity of systemic inflammation was determined using a panel of cytokines/chemokines, and the severity of LPS-induced organ injury was quantified by measurement of classical biochemical biomarkers.

## Methods

### LPS-induced model of endotoxemia in young and aged mice

In pilot studies, the dose of lipopolysaccharides (LPS) from *Salmonella typhosa* (ATCC 10749 source strain) was tested in the range 3–10 mg/kg (Supplementary Information) to induce significant endothelial response without a risk of high mortality in aged animals. Based on these findings, a relatively low dose of LPS (3 mg/kg) was chosen for the current study.

C57BL/6 mice were weighed at the beginning of each experimental day. Young (3-month-old) and aged (18-month-old) mice received a single intraperitoneal administration of 0.9% NaCl (0 h) or LPS at a dose of 3 mg/kg, and LPS response was analyzed 1 h, 2 h, 3 h, 12 h, and 24 h after LPS, and at the end of each time point, mice were euthanized (100 mg/kg ketamine + 10 mg/kg xylazine, *i.p.*). Then, blood was drawn from the heart, collected in tubes containing 10% solution of K2EDTA (Aqua-Med, Łódź, Poland, 1.6 μL of 10% K2EDTA solution/100 μL of blood), and centrifuged for 10 min (664 × g). The blood designated for biomarker analysis was supplemented with MS-SAFE protease and phosphatase inhibitor cocktail (PIC; Sigma-Aldrich, St. Louis, MO, USA) in a ratio of 100:1. The obtained plasma was collected, divided into aliquots, and stored at − 80 °C until analysis. All selected tissues such as lungs, liver, kidney, spleen, and brain were washed free of blood with saline, and excess liquid was drained using lint-free wipes. They were then weighed and stored for analysis.

The severity of lung edema induced by LPS was accessed by the calculation of W/D ratio [38–40]. After finishing the experiment, the right part of the lung was removed, and its wet weight was determined. Then, it was dried using FreeZone lyophilizer (Labconco, Kansas City, MO, USA) at − 40 °C for 48 h. The dry weight was determined, and the W/D ratios were calculated.

The animals were housed in collective cages, in a room with constant environmental conditions (22–25 °C, 65–75% humidity, and a 12-h light/dark cycle). Mice had ad libitum access to daily provided chow diet and water. All of the experiments were approved by the II Local Ethical Committee on Animal Testing in the Institute of Pharmacology, Polish Academy of Sciences (Krakow, Poland), and were in accordance with the Guide for the Care and Use of Laboratory Animals of the National Academy of Sciences [41], as well as the Guidelines for Animal Care and Treatment of the European Community.

### Assessment of endothelium-dependent response to acetylcholine and endothelium-independent response to sodium nitroprusside in vivo by magnetic resonance imaging (MRI)

To assess endothelial function in vivo, MRI with the 9.4 T scanner (BioSpec 94/20 USR, Bruker, Germany) was used as described previously [13, 42–47]. Briefly, mice were imaged in the supine position under stable anesthesia (isoflurane (1.5 vol%) in oxygen and air (1:2) mixture) and heart function; respiration and body temperature (maintained at 37 °C using circulating warm water) were monitored using a Monitoring and Gating System (SA Inc., Stony Brook, NY, USA).

The endothelial function in vivo was assessed as an endothelium-dependent response to acetylcholine (Ach: 50 μL, 16.6 mg/kg, *i.p.*), while endothelium-independent response was induced by sodium nitroprusside (SNP, 1 mg/kg, *i.v.*). Measurements were performed 2 h and 12 h after LPS administration. The vasomotor responses were examined in the abdominal (AA) and thoracic aorta (TA) by comparing two, time-resolved 3D images of the vessels prior to and 30 min after Ach or SNP administration. Vascular responses were expressed as a percentage of changes in the vessel volume after Ach or SNP administration and defined as vasodilation and vasoconstriction for positive and negative values, respectively. The methodology for vascular response analysis based on MRI images was described in details in our previous work [48]. Images were acquired using the cine IntraGate™ FLASH 3D sequence and reconstructed with the IntraGate 1.2.b.2 macro (Bruker, Billerica, MA, USA) with imaging parameters and imaging analysis as described previously [42, 43, 48].

### Assessment of a biomarker panel reflecting various aspect of endothelial function by microLC-MS/MS

To characterize in more detail the profile of endothelial response to LPS, a comprehensive panel of endothelium-associated proteins was quantified in murine plasma using a targeted proteomic method as described elsewhere [42, 43, 49–51]. The panel included 20 protein/peptide biomarkers with 12 of them previously validated [52, 53] and 8 of them validated here (see below). The panel included biomarkers of *glycocalyx disruption*: syndecan-1 (SDC-1) and endocan (ESM-1); *endothelial inflammation*: the soluble vascular cell adhesion molecule 1 (sVCAM-1), the soluble intercellular adhesion molecule 1 (sICAM-1), the soluble form of E-selectin (sE-sel), and the soluble form of P-selectin (sP-sel); *endothelial permeability*: angiopoietin 1 (Angpt-1), angiopoietin 2 (Angpt-2), the soluble form of transmembrane tyrosine-protein kinase receptor for Angpt-1, Angpt-2 and Angpt-4 (sTie-2), and the soluble form of fms-like tyrosine kinase (sFLT-1); *hemostasis*: von Willebrand factor (vWF), tissue plasminogen activator (t-PA), and plasminogen activator inhibitor 1 (PAI-1), thrombin activatable fibrinolysis inhibitor (TAFI), thrombospondin 1 (THBS-1), and the soluble form of thrombomodulin (sTM); and *other molecules* such as adrenomedullin (ADM), adiponectin (ADN), annexin A5 (ANXA5), and myelin-associated glycoprotein (MAG).

Briefly, the murine plasma was subjected to proteolytic digestion using porcine trypsin to achieve unique and reproducible peptide sequences, applied as the surrogates of the proteins suitable for microLC-MS/MS analyses as described previously in details for 12 initial biomarkers in the panel [52–55]. For quantitative measurements, an UPLC Nexera system (Shimadzu, Kyoto, Japan) connected to a highly sensitive mass spectrometer (QTrap 5500, Sciex, Framingham, MA, USA) was used.

### Validation of eight newly added protein biomarkers to targeted proteomic-based panel of biomarkers of endothelial dysfunction

To measure the additional eight biomarkers, specific tryptic peptides generated by trypsin digestion in mouse plasma were identified and selected as surrogate peptides for sP-sel, Angpt-1, sTie-2, TAFI, THBS-1, ANXA5, MAG and sTM. The chosen peptides were quantified by monitoring several MRM transitions. Labelled peptides were used as the internal standards for protein quantification by microLC/MS-MRM. In this study, the Analyst software version 1.6.2 (SCIEX, Framingham, MA, USA) was used.

The chromatographic and mass spectrometric conditions, for the panel included 20 protein/peptide biomarkers with 12 of them previously validated and 8 of them validated here, are described in the Supplementary Information and listed in Suppl. Tables 1.1–5.1.

The newly added, 8 protein biomarkers and their stable isotope-labelled internal standards were validated with respect to linearity, accuracy, precision, recovery, and matrix effect (Supplementary Information).

For identification and quantification of selected proteins, specific amino acid sequences as the surrogates of total proteins were chosen based on bioinformatic tools such as Peptide Cutter (Expasy), UniProt, Peptide Atlas and Gene Pattern ESP Predictor.

Surrogate peptides and their stable isotope-labelled internal standards (SISs) originally packed to the vial in the form of a lyophilizate weighing 1 mg, provided with quality certificates, were synthesized by Innovagen AB (Lund, Sweden). The quality control analyses of all peptides were performed with the aid of HPLC, nano-spray MS, and LC–MS/MS methods. The purity of the peptides was ≥ 95%. The synthesized, specific peptides used as the surrogates of total proteins were chosen to share the sequences between mice and humans, if possible. Sequences of peptides and their SISs are presented in Suppl. Table 1.1.

To confirm the effectiveness of the re-validated method, western blots were performed for selected proteins such as sTM, ANXA5, and sTie-2. The methodological data and obtained results are presented in Supplementary Information and in Suppl. Figure 6, respectively.

### Blood count and biochemical biomarkers of organ injury

Whole blood was used for determination of the following hematological parameters: white blood cells (WBC), count of red blood cells (RBC) and platelets (PLT), hemoglobin (HGB), hematocrit (HCT), mean cell volume (MCV), mean corpuscular hemoglobin (MCH), and mean corpuscular hemoglobin concentration (MCHC) with the aid of an ABC Vet analyzer (Horiba, Kyoto, Japan). Plasma creatinine, alanine transaminase (ALT), aspartate transaminase (AST), urea, and glucose were measured using a Pentra 400 (Horiba, Kyoto, Japan).

### Measurement of myeloperoxidase activity in lung homogenates

Mouse lungs were homogenized in PBS (1 mL/100 mg tissue) using automatic Precellys Evolution homogenizer (three cycles: 7500 rpm × 30 s/30 s brake/4 °C; Bertin Technologies SAS, France) and centrifuged (5000 × g/5 min/4 °C). Diluted (100 ×) supernatant samples were used for the analysis. Quantitative determination of myeloperoxidase (MPO) concentration in mouse lung homogenates was performed using commercially available ELISA assay (CSB-E08723m; Cusabio, Houston, TX, USA) following the manufacturer’s instructions. Accurate MPO levels measured in lung tissue was performed using a Synergy H4 Hybrid Reader (BioTek Instruments, Männedorf, Switzerland) and finally calculated as pg/mg of the right lung.

### Assessment of systemic inflammation based on serum amyloid A and panel of 23 cytokines/chemokines in mouse plasma

Serum amyloid A (SAA) was determined with the aid of Phase SAA Murine Assay Kit (TP-802 M; Tridelta Development Ltd, Maynooth, Ireland) using Synergy H4 Hybrid Reader (BioTek Instruments, Männedorf, Switzerland). The concentration of 23 cytokines/chemokines in the plasma was measured with Bio-Plex Pro™ Mouse Cytokine 23-plex Assay Kit (M60009RDPD; Bio-Rad Laboratories, Ltd., Hercules, CA, USA) on the Bio-Plex 200 System (Bio-Rad Laboratories, Ltd., Hercules, CA, USA) according to manufacturer’s instructions.

The panel of measured cytokines/chemokines included: interleukin-1α (IL-1α), interleukin-1β (IL-1β), interleukin-2 (IL-2), interleukin-3 (IL-3), interleukin-4 (IL-4), interleukin-5 (IL-5), interleukin-6 (IL-6), interleukin-9 (IL-9), interleukin-10 (IL-10), interleukin-12(p40) (IL-12(p40)), interleukin-12(p70) (IL-12(p70)), interleukin-13 (IL-13), interleukin-17α (IL-17α), eotaxin, granulocyte colony-stimulating factor (G-CSF), granulocyte–macrophage colony-stimulating factor (GM-CSF), interferon gamma (IFN-γ), keratinocyte-derived cytokine (KC), monocyte chemoattractant protein-1 (MCP-1), macrophage inflammatory protein-1α (MIP-1α), macrophage inflammatory protein-1β (MIP-1β), chemokine named as RANTES (Regulated on Activation, Normal T-cell Expressed and Secreted), and tumor necrosis factor-α (TNF-α).

### Determination of nitric oxide metabolites in plasma

For nitrite (NO_2_^−^) and nitrate (NO_3_^−^) determination, plasma was pelleted with methanol (MeOH; Sigma-Aldrich, St. Louis, MO, USA), centrifuged (14,462 × g, 10 min) and the supernatant was used for analysis immediately after sample preparation. For this type of analysis, a sensitive HPLC-based technique was used (ENO-20 NOx Analyzer; EiCom, Kyoto, Japan), as described previously [52, 56–58].

### Data analysis

Data were presented as the mean and standard error of mean (mean ± SEM). After verifying data normality (Shapiro–Wilk test) and the equality of variances (Brown–Forsythe test), parametric (ANOVA tests) or non-parametric (Kruskal–Wallis tests) calculations were performed. Student’s *t*-test or Mann–Whitney *U* test was used for point-to-point comparisons between young and aged mice. The results were evaluated with GraphPad Prism 9.1.1 (San Diego, CA, USA), with *p* < 0.05 considered statistically significant.

## Results

### Comparison of the functional endothelial response to LPS in aged vs. young C57BL/6 mice assessed by MRI in vivo

**Fig. 1:**
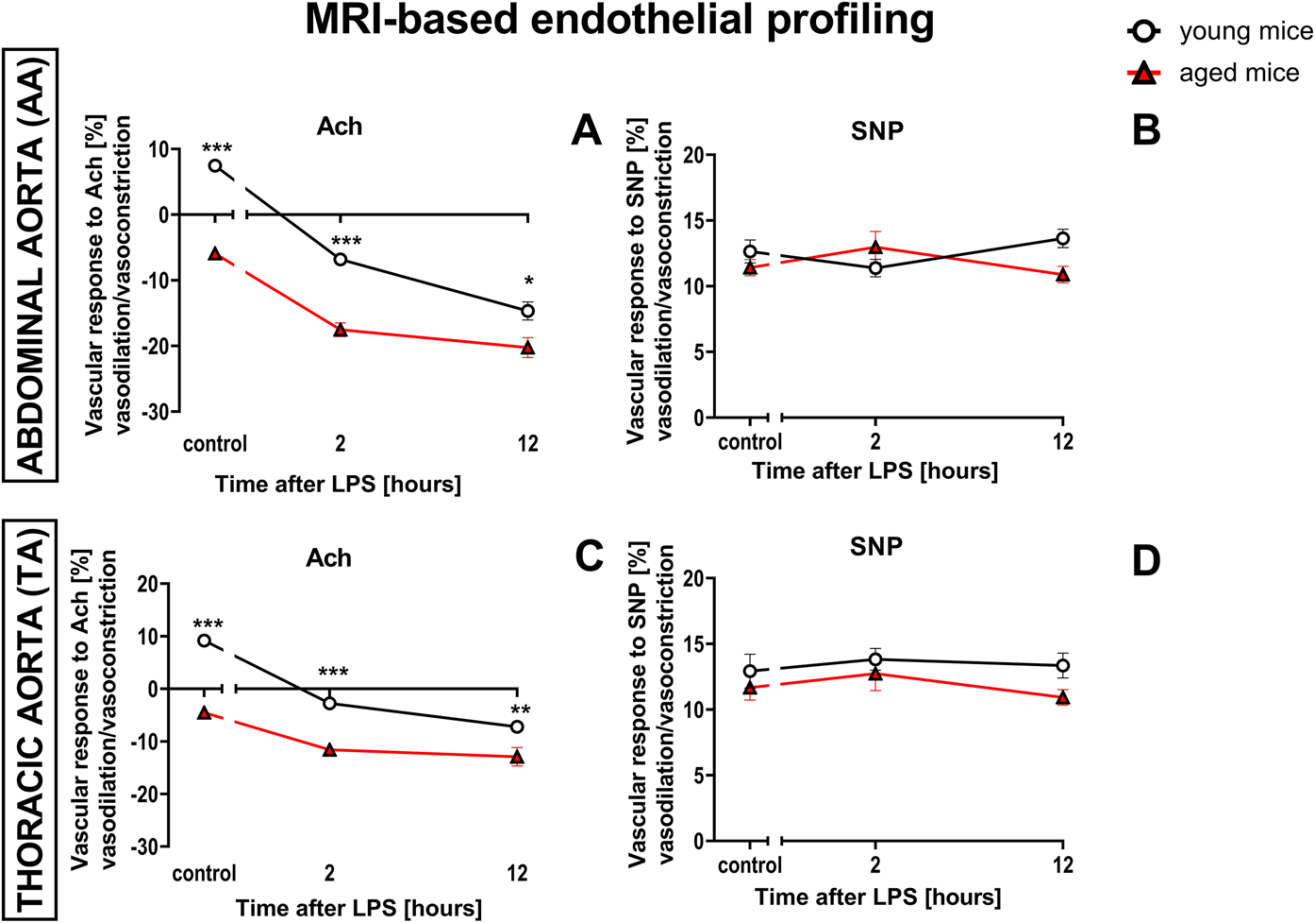
Endothelial dysfunction induced by LPS (3 mg/kg) in young and aged C57BL/6 mice. Changes in the end-diastolic volume of the AA (**A, B**) and TA (**C, D**) 30 min after Ach (**A, C**; *n* = 5–6) or SNP (**B, D**; *n* = 5–6) administration, in young (3-month-old), and aged (18-month-old) C57BL/6 mice were measured in vivo by MRI. The results are presented as means (–) ± SEM. *, **, and *** indicate statistically significant difference between young mice and aged mice at the same timepoint with *p* < 0.05, *p* < 0.01, and *p* < 0.001, respectively. Acetylcholine (Ach), the abdominal part of the aorta (AA), sodium nitroprusside (SNP), the thoracic part of the aorta (TA)

LPS (3 mg/kg, *i.p.*) induced endothelial dysfunction that was more pronounced in aged mice (18-month-old), as compared to young mice (3-month-old), but basal endothelial function before LPS was also compromised in aged mice as compared with young mice. In aged mice, under baseline conditions, there was a paradoxical vasoconstriction in response to Ach in the abdominal (AA) and thoracic (TA) parts of the aorta (− 5.9% and − 4.5%, respectively), while in young mice under baseline conditions, Ach induced a vasodilation in the AA and TA (7.6% and 9.2%, respectively) (Fig. 1A, C). SNP in control conditions induced vasodilation of similar magnitude in young and aged mice (AA: 12.6% vs. 11.4%; TA: 12.9% vs. 11.7%, respectively) (Fig. 1B, D). As early as 2 h after LPS injection (3 mg/kg, *i.p.*) aged mice responded with further amplification of Ach-induced vasoconstriction in the AA and TA (− 17.5% for the AA and − 11.6% for TA). Twelve hours after LPS, Ach-induced vasoconstriction in the AA and TA was also more pronounced in aged mice as compared with young mice (− 20.2% for the AA and − 12.9% for TA (Fig. 1A, C). In contrast to the response to Ach, LPS did not induce any changes in endothelium-independent response to SNP in the AA (Fig. 1B) and TA (Fig. 1D), neither 2 h nor 12 h after LPS administration.

### Comparison of the profile of endothelial response to LPS in aged vs. young C57BL/6 mice based on panel of endothelial biomarkers measured by microLC-MS/MS

LPS at a dose of 3 mg/kg induced a pronounced early and time-dependent changes in levels of various biomarkers in contrast to higher (5 mg/kg and 10 mg/kg) doses of LPS (Supplementary Information). LPS (3 mg/kg, *i.p.*) induced changes in measured biomarkers reflected various aspect of endothelial phenotype, with some changes that did not differ between aged and young mice (sVCAM-1, sICAM-1, sE-sel, sP-sel, PAI-1, t-PA, vWF, sFLT-1), some that showed quantitative difference (sTie-2, ANXA5, ADM, ADN and MAG), and some displaying a clear difference in response profile (SDC-1, ESM-1, Angpt-2, sTM, TAFI, THBS-1).

**Fig. 2:**
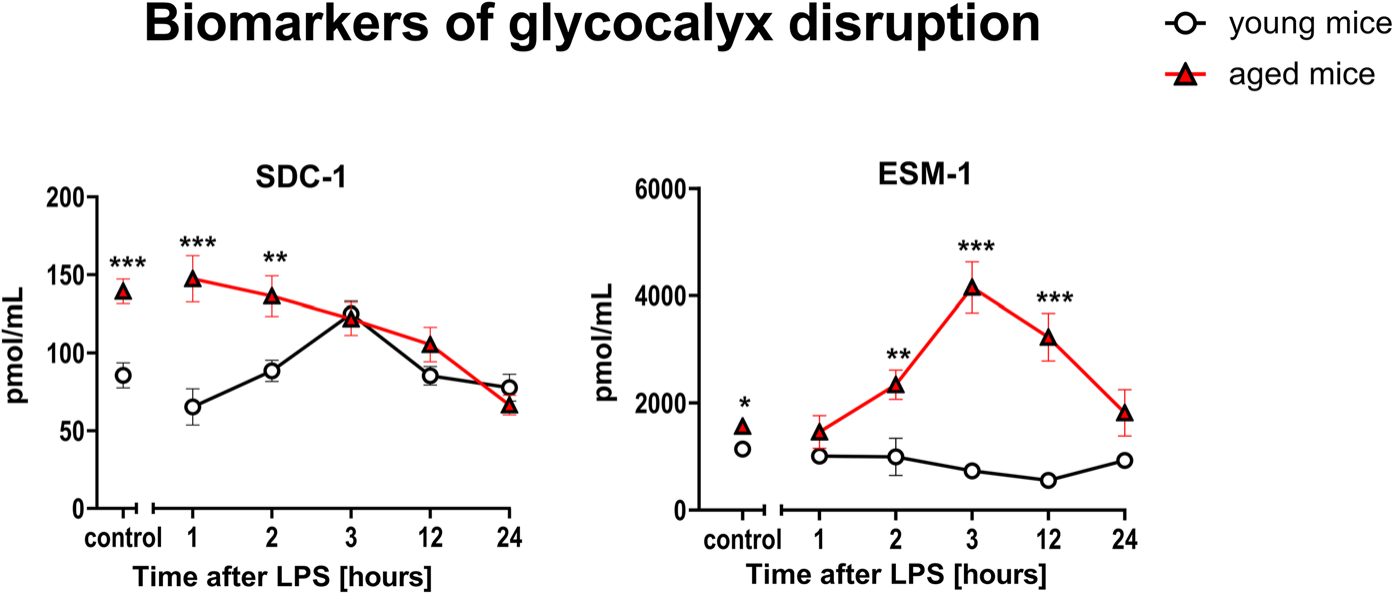
Changes in the plasma concentration of biomarkers reflecting glycocalyx disruption in endotoxemia induced by LPS (3 mg/kg) in young and aged C57BL/6 mice. Plasma protein biomarkers characterizing glycocalyx dysregulation were measured by microLC/MS-MRM (*n* = 8–10). The results are presented as means (–) ± SEM. *, **, and *** indicate statistically significant difference between young mice and aged mice at the same timepoint with *p* < 0.05, *p* < 0.01, and *p* < 0.001, respectively. Endocan (ESM-1), syndecan-1 (SDC-1)

In the early phase of endotoxemia (1–3 h after LPS), the response of SDC-1, representative of glycocalyx injury, was different in aged mice as compared to young mice, and was characterized by initially high SDC-1 in aged mice with a progressive fall after LPS, while in young mice, there was a modest increase in SDC-1 after LPS (Fig. 2). Another biomarker for glycocalyx injury, ESM-1 displayed consistently higher plasma levels in aged mice 2–12 h after LPS, while there was no changes in ESM-1 plasma levels after LPS in young mice (Fig. 2).

**Fig. 3:**
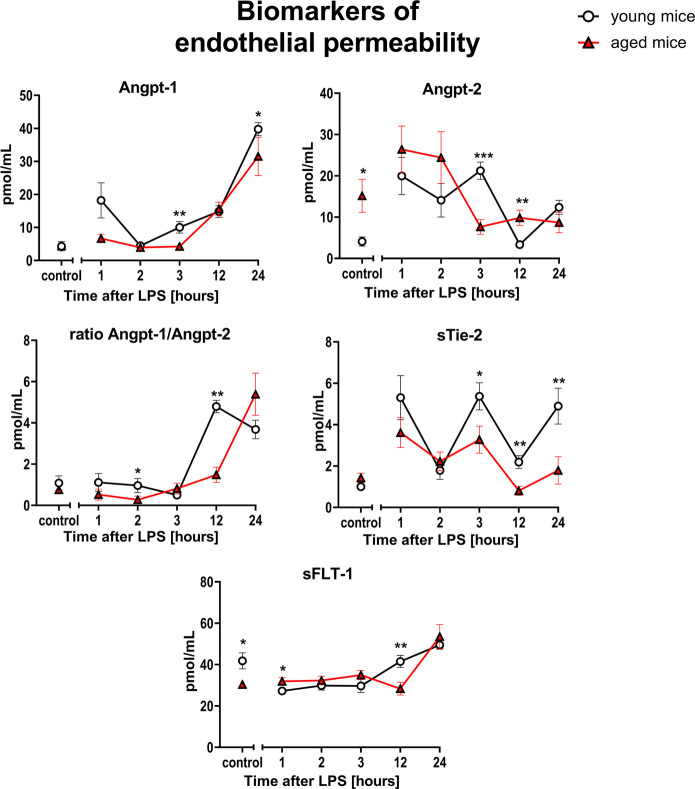
Changes in the plasma concentration of biomarkers reflecting endothelial permeability in endotoxemia induced by LPS (3 mg/kg) in young and aged C57BL/6 mice. Plasma protein biomarkers characterizing endothelial permeability were measured by microLC/MS-MRM (*n* = 8–10). The results are presented as means (–) ± SEM. *, **, and *** indicate statistically significant difference between young mice and aged mice at the same timepoint with *p* < 0.05, *p* < 0.01, and *p* < 0.001, respectively. Angiopoietin 1 (Angpt-1), angiopoietin 2 (Angpt-2), the soluble form of transmembrane tyrosine-protein kinase receptor for Angpt-1, Angpt-2 and Angpt-4 (sTie-2), the soluble form of fms-like tyrosine kinase (sFLT-1)

Amongst the biomarkers of endothelial permeability, Angpt-1 was lower (1–24 h after LPS) and Angpt-2 was higher in aged than in young mice at several timepoints after LPS (1 h, 2 h, and 12 h). The Angpt-1/Angpt-2 ratio also tended to be lower in aged mice, with statistically significant difference observed 12 h after LPS. At this timepoint, sTie-2 was also significantly lower in aged mice than in young mice (Fig. 3).

**Fig. 4:**
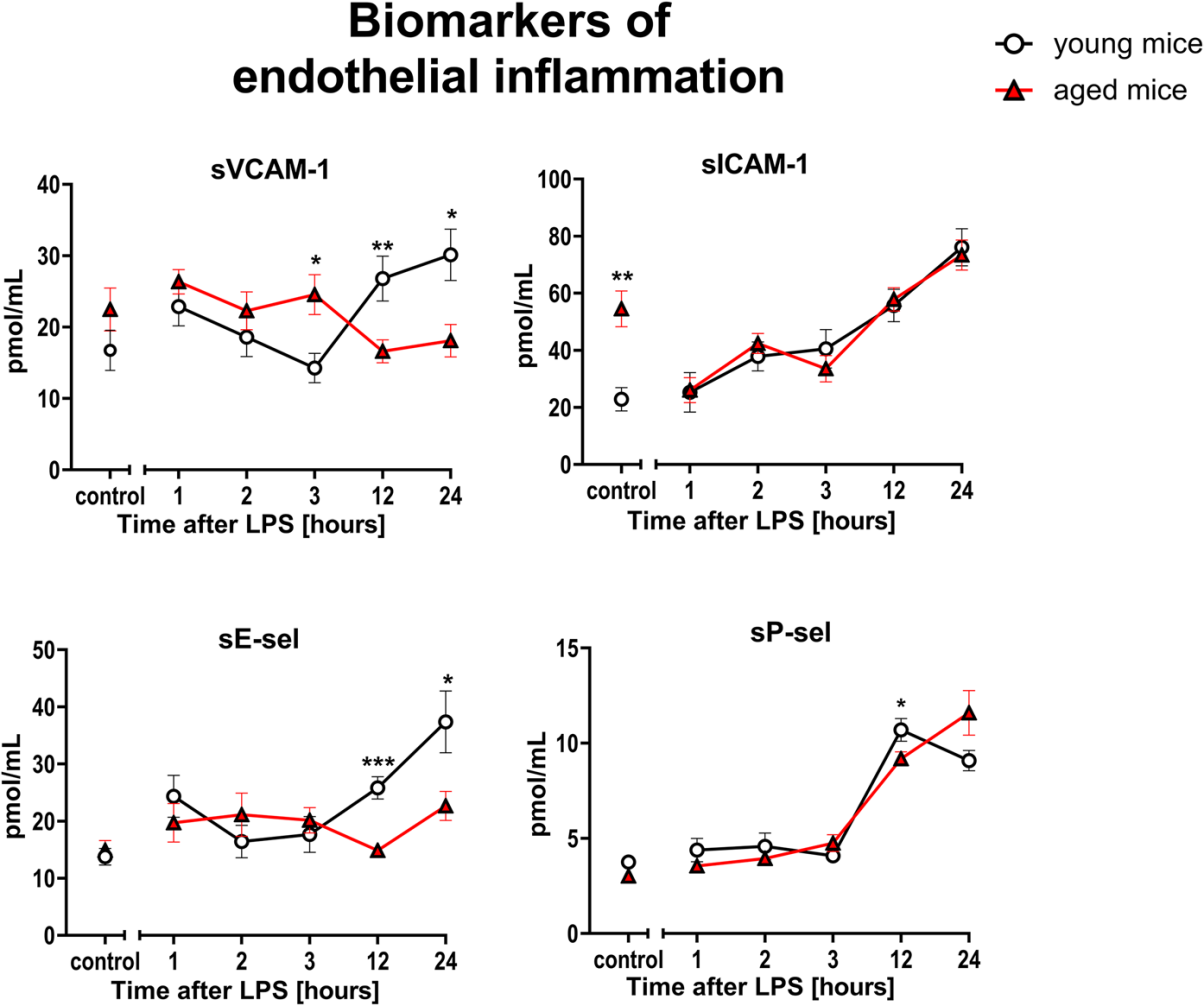
Changes in the plasma concentration of biomarkers reflecting endothelial inflammation in endotoxemia induced by LPS (3 mg/kg) in young and aged C57BL/6 mice. Plasma protein biomarkers characterizing endothelial inflammation were measured by microLC/MS-MRM (*n* = 8–10). The results are presented as means (–) ± SEM. *, **, and *** indicate statistically significant difference between young mice and aged mice at the same timepoint with *p* < 0.05, *p* < 0.01, and *p* < 0.001, respectively. The soluble vascular cell adhesion molecule 1 (sVCAM-1), the soluble intercellular adhesion molecule 1 (sICAM-1), the soluble form of E-selectin (sE-sel), the soluble form of P-selectin (sP-sel)

**Fig. 5:**
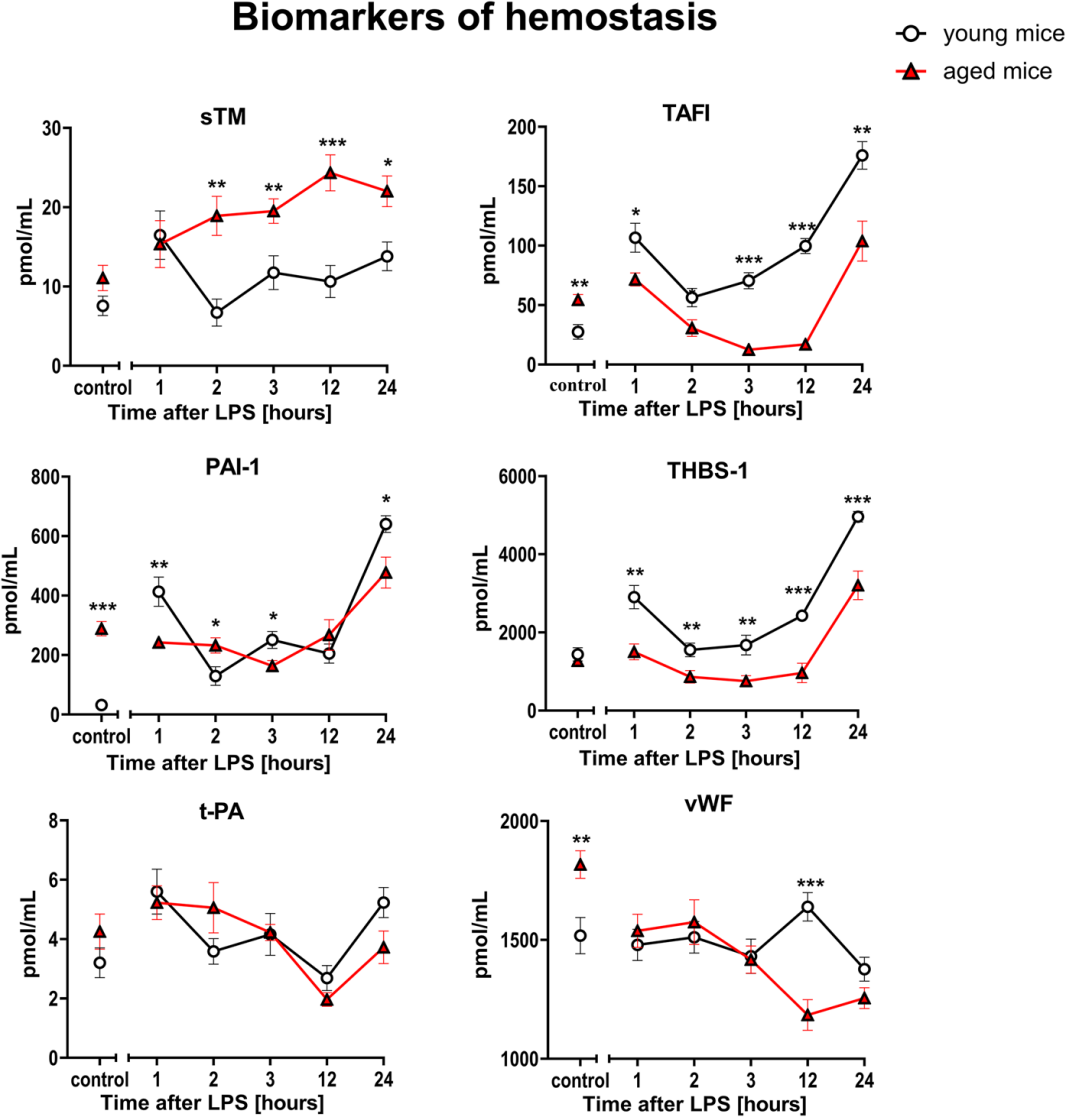
Changes in the plasma concentration of biomarkers reflecting hemostasis in endotoxemia induced by LPS (3 mg/kg) in young and aged C57BL/6 mice. Plasma protein biomarkers characterizing endothelial hemostasis were measured by microLC/MS-MRM (*n* = 8–10). The results are presented as means (–) ± SEM. *, **, and *** indicate statistically significant difference between young mice and aged mice at the same timepoint with *p* < 0.05, *p* < 0.01, and *p* < 0.001, respectively. The soluble form of thrombomodulin (sTM), thrombin activatable fibrinolysis inhibitor (TAFI), plasminogen activator inhibitor 1 (PAI-1), thrombospondin 1 (THBS-1), tissue plasminogen activator (t-PA), von Willebrand factor (vWF)

Interestingly, biomarkers of endothelial inflammation (sVCAM-1, sICAM-1, sE-sel, sP-sel) (Fig. 4) and classical hemostasis biomarkers (PAI-1, t-PA, vWF) (Fig. 5) displayed similar responses to LPS in aged and young mice.

**Fig. 6:**
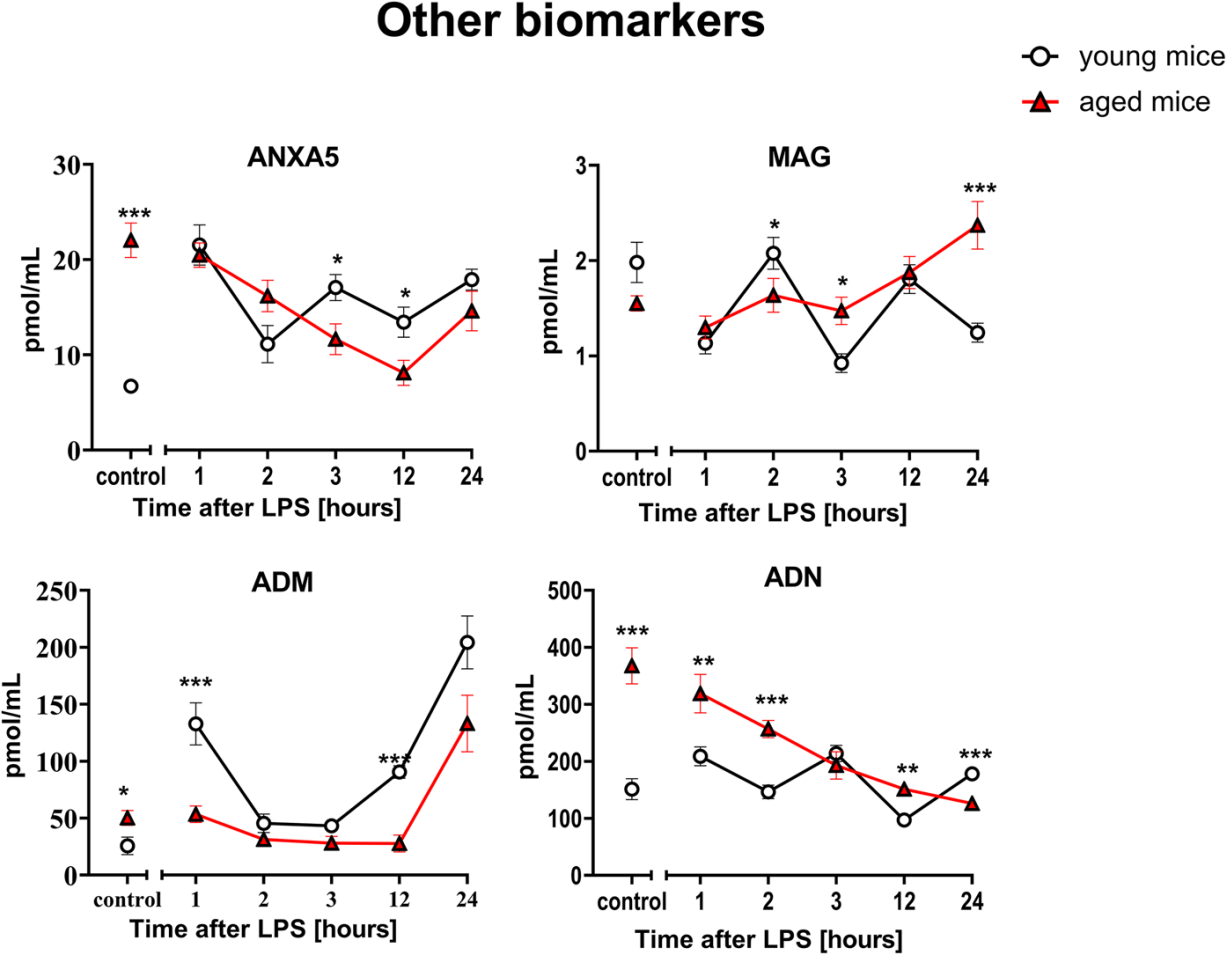
Changes in the plasma concentration of other biomarkers in endotoxemia induced by LPS (3 mg/kg) in young and aged C57BL/6 mice. Plasma protein/peptide biomarkers characterizing other endothelium-related biomarkers were measured by microLC/MS-MRM (*n* = 8–10). The results are presented as means (–) ± SEM. *, **, and *** indicate statistically significant difference between young mice and aged mice at the same timepoint with *p* < 0.05, *p* < 0.01, and *p* < 0.001, respectively. Annexin A5 (ANXA5), myelin-associated glycoprotein (MAG), adrenomedullin (ADM), adiponectin (ADN)

On the other hand, a distinct, age-dependent pattern of endothelial response was also observed for selected biomarkers specific for endothelial hemostasis (sTM, TAFI and THBS-1) (Fig. 5). The sTM response to LPS was more pronounced resulting in higher plasma levels in aged mice as compared to young mice throughout the observation period (1–24 h after LPS), whereas TAFI and THBS-1 responses to LPS were blunted in aged mice as compared with young mice and displayed lower plasma levels after LPS in aged vs. young mice. Some differences were noted for ADM and MAG at various timepoints after LPS (Fig. 6).

The data obtained from targeted proteomic analyses were also analyzed using principal component analysis (PCA analysis) and presented in the Suppl. Figure 4.

### Comparison of the systemic inflammation in response to LPS in aged vs. young C57BL/6 mice

**Fig. 7:**
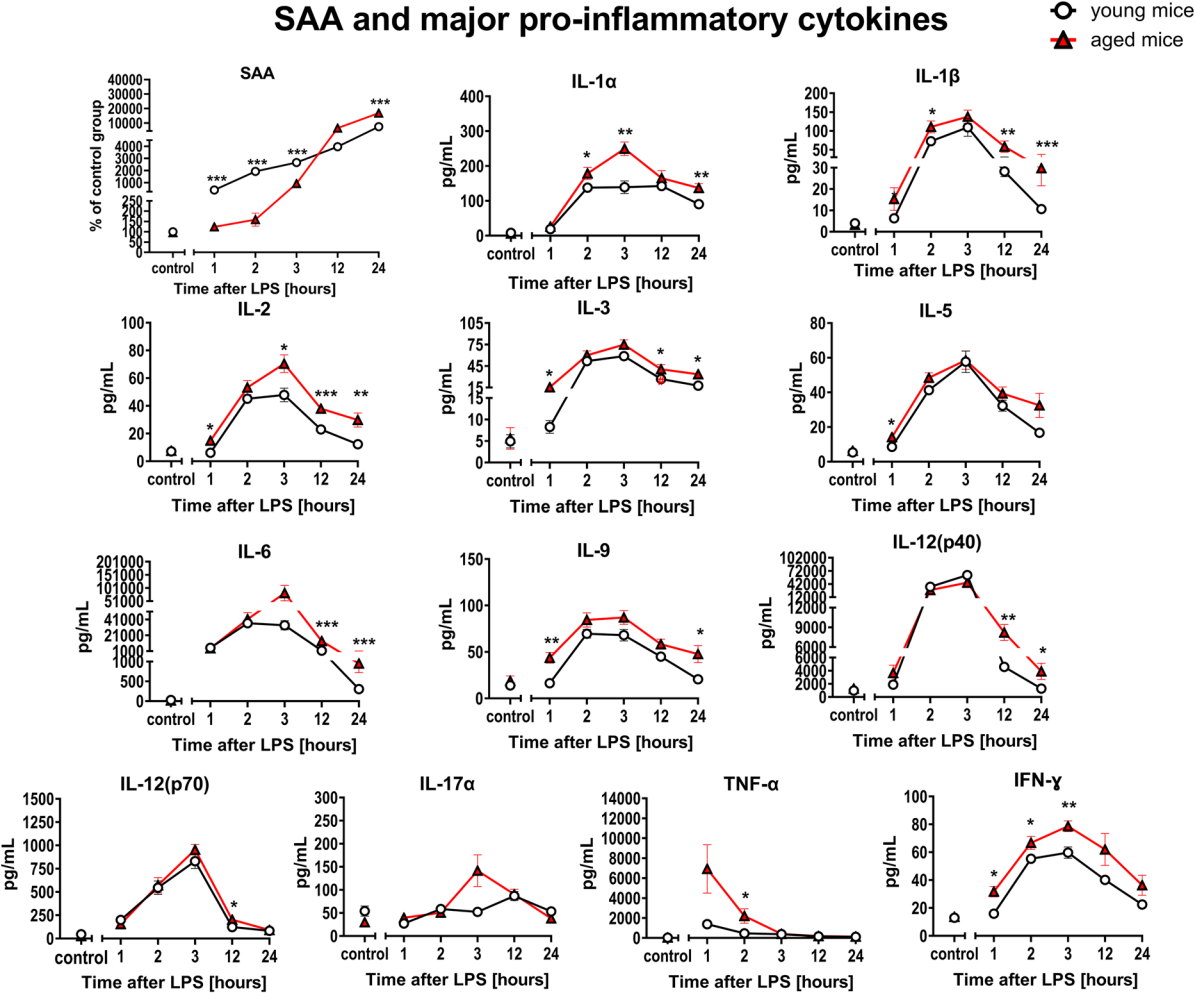
Changes in plasma concentration of SAA and major pro-inflammatory cytokines in endotoxemia induced by LPS (3 mg/kg) in young and aged C57BL/6 mice. SAA (*n* = 8–10) and major cytokines with pro-inflammatory (*n* = 6–18) action were measured in mouse plasma using single or multiplex immunoassays. The results are presented as means (–) ± SEM. *, **, and *** indicate statistically significant difference between young mice and aged mice at the same timepoint with *p* < 0.05, *p* < 0.01, and *p* < 0.001, respectively. Interleukin-1α (IL-1α), interleukin-1β (IL-1β), interleukin-2 (IL-2), interleukin-3 (IL-3), interleukin-5 (IL-5), interleukin-6 (IL-6), interleukin-9 (IL-9), interleukin-12(p40) (IL-12(p40)), interleukin-12(p70) (IL-12(p70)), interleukin-17α (IL-17α), interferon gamma (IFN-γ) and tumor necrosis factor-α (TNF-α)

**Fig. 8:**
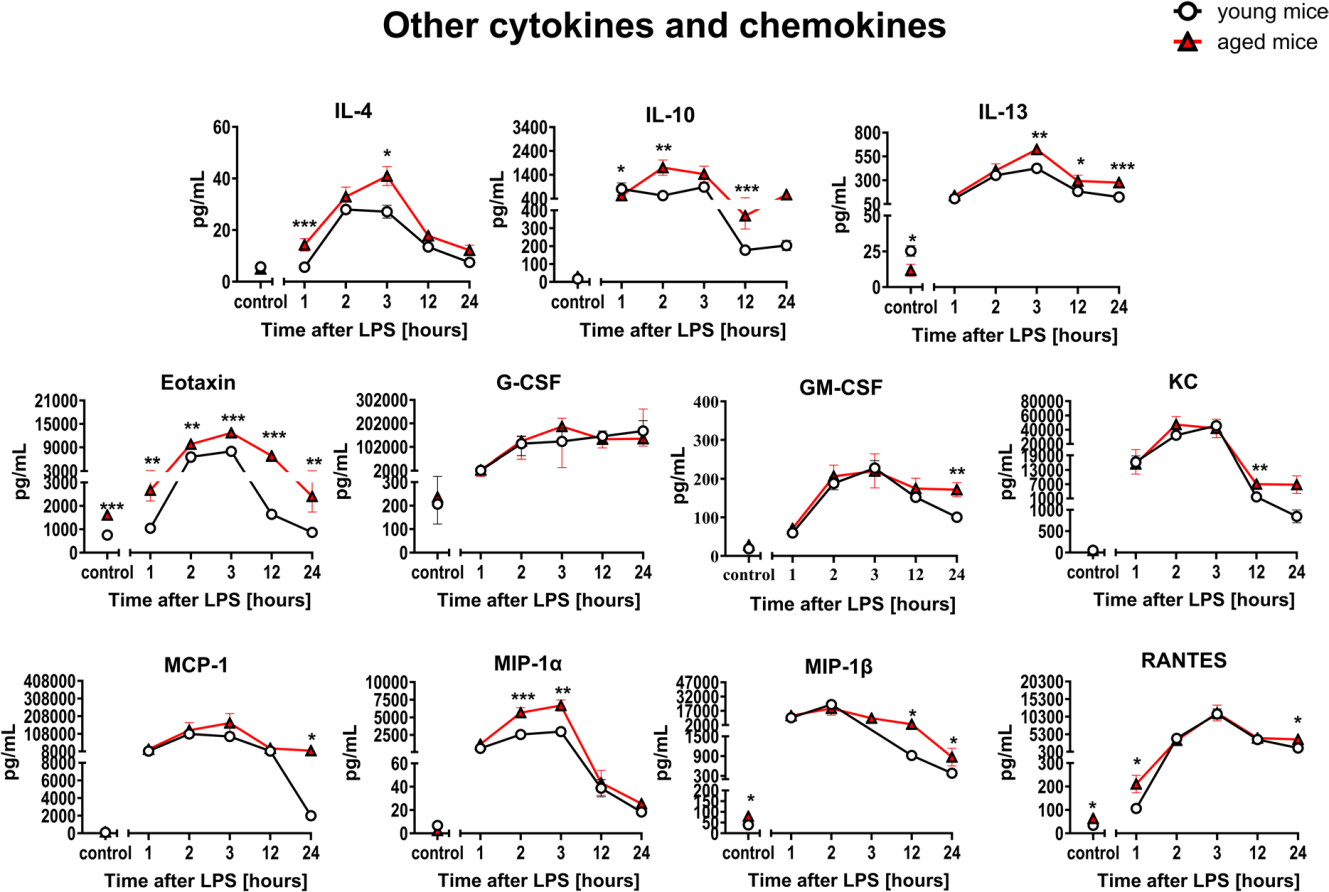
Changes in plasma concentration of other cytokines and chemokines in endotoxemia induced by LPS (3 mg/kg) in young and aged C57BL/6 mice. Additional cytokines and chemokines (*n* = 6–18) were measured in mouse plasma using multiplex immunoassays. The results are presented as means (–) ± SEM. *, **, and *** indicate statistically significant difference between young mice and aged mice at the same timepoint with *p* < 0.05, *p* < 0.01, and *p* < 0.001, respectively. Eotaxin, granulocyte colony-stimulating factor (G-CSF); granulocyte–macrophage colony-stimulating factor (GM-CSF); interleukin-4 (IL-4); interleukin-10 (IL-10); interleukin-13 (IL-13); keratinocyte-derived cytokine (KC); monocyte chemoattractant protein-1 (MCP-1); macrophage inflammatory protein-1α (MIP-1α); macrophage inflammatory protein-1β (MIP-1β); chemokine named as RANTES (Regulated on Activation, Normal T-cell Expressed and Secreted)

To verify whether distinct endothelial response to LPS in aged as compared with young mice was related to differences in the intensity of systemic inflammation, SAA and 23 cytokines/chemokines were measured (Figs. 7 and 8). In young mice, the systemic inflammatory response evidenced by high concentration of SAA (Fig. 7) occurred more rapidly compared to that in aged mice, whereas in aged mice developed slower but culminated in higher end-points plasma values (12 h and 24 h after LPS) as compared with young mice. Moreover, in both young and aged mice, white blood cell and platelet counts decreased in response to LPS, with the similar pattern of responses to LPS, but with higher initial PLT count in aged mice as compared with young mice (Suppl. Table 1). Furthermore, NOS2 induction evidenced by increased plasma concentration of NO_3_^−^ (Suppl. Table 1) was also similar in aged and young mice with initially higher plasma NO_3_^−^ levels in aged vs. young mice. In contrast to NO_3_^−^, plasma concentration of NO_2_^−^ did not significantly change after LPS and the pattern was again similar in young and aged mice (data not shown). Interestingly, changes in plasma glucose levels were also noticed to be more intense in aged mice as compared with young mice during the endotoxemia development (Suppl. Table 1).

The panel of cytokines/chemokines measured in aged and young mice after LPS included pro-inflammatory mediators: IL-1α, IL-1β, IL-2, IL-3, IL-5, IL-6, IL-9, IL-12(p40), IL-12(p70), IL-17α, eotaxin, G-CSF, GM-CSF, IFN-γ, KC, MCP-1, MIP-1α, MIP-1β, RANTES, and TNFα, as well as anti-inflammatory mediators: IL-4, IL-10, and IL-13 (Figs. 7 and 8).

The majority of the 23 cytokines/chemokines measured in aged and young mice did not show significant age-dependent differences in response to LPS with the exception of IL-1β, IL-2, and eotaxin that displayed significantly different plasma concentration in at least four out of five timepoints after LPS administration.

Similar conclusions were drawn based on PCA using determined data from a panel of 23 cytokines/chemokines. There were some other less pronounced changes that differentiated LPS response in aged and young mice as shown in Figs. 7 and 8 and in Suppl. Figure 5.

### Comparison of biomarkers of organ injury in response to LPS in aged vs. young C57BL/6 mice

To verify whether the altered endothelial response profile to LPS administered at a relatively low dose of 3 mg/kg *i.p.* was associated with more pronounced organ damage in aged mice compared with young mice, biochemical biomarkers of organ damage, such as AST, ALT, creatinine and urea, were analyzed.

**Fig. 9:**
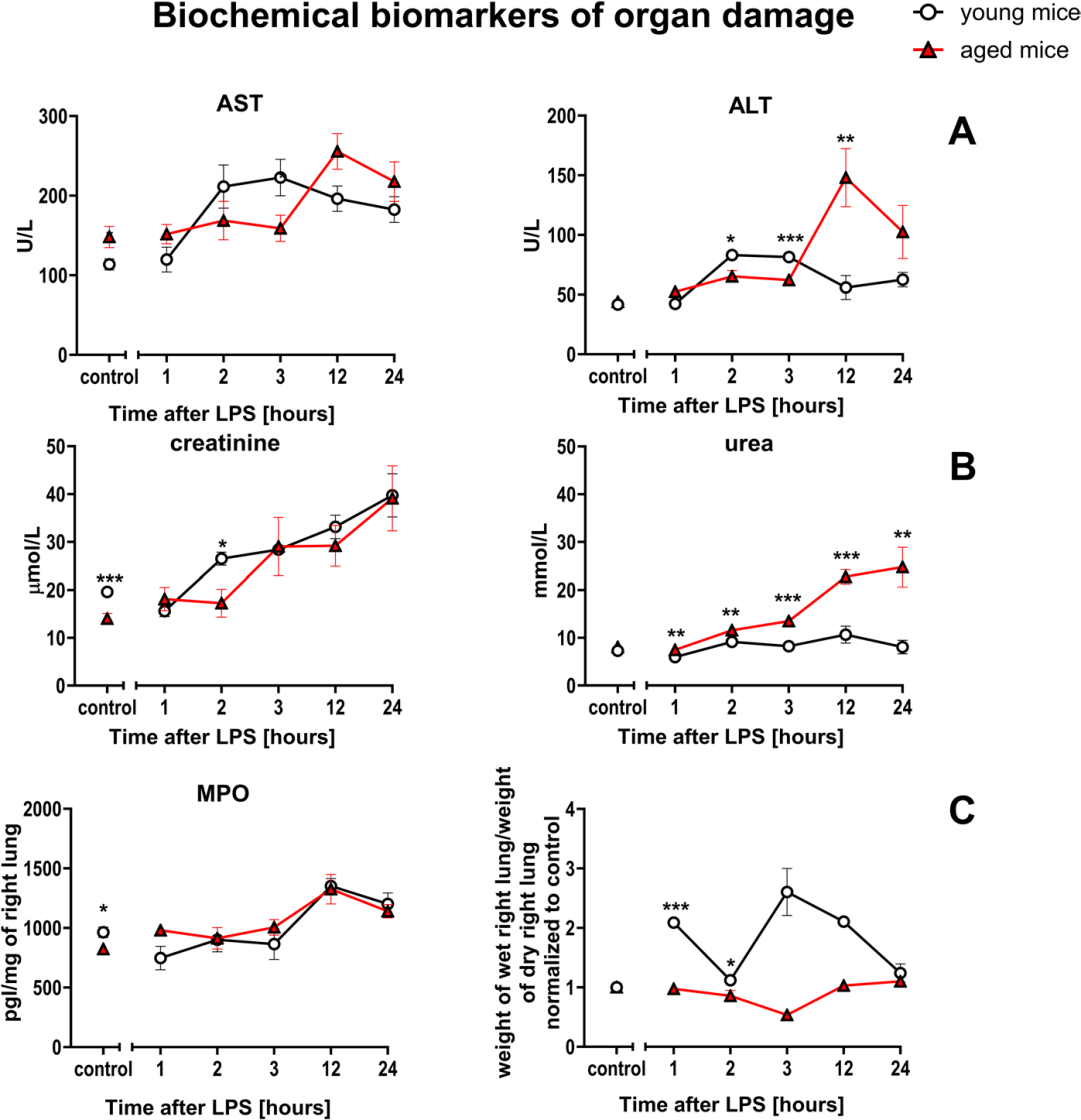
Changes in the plasma concentration of biochemical biomarkers reflecting organ damage in endotoxemia induced by LPS, (3 mg/kg) in young and aged mice. Liver injury was assessed by AST, ALT (**A**; *n* = 4–11), kidneys by creatinine, urea (**B**; *n* = 4–11), and lungs by MPO activity and dry/wet ratio (**C**; *n* = 4–11). The results are presented as means (–) ± SEM. *, **, and *** indicate statistically significant difference between young mice and aged mice at the same timepoint with *p* < 0.05, *p* < 0.01, and *p* < 0.001, respectively. Alanine transaminase (ALT), aspartate transaminase (AST), myeloperoxidase (MPO)

As shown in Fig. 9, there was a more pronounced response to LPS in aged mice as compared with young mice for ALT and urea, but not for AST and creatinine. MPO activity (in lung homogenates) or lung edema (Fig. 9C) calculated based on ‘dry/wet ratio’ also did not revealed more pronounced lung injury in aged than in young mice. Similarly, total organ weights showed no significant differences between the response of aged and young mice to LPS (Suppl. Figure 1).

## Discussion

The present study demonstrates that aging is associated with a specific endothelial response profile to LPS. In aged mice, LPS not only caused a more pronounced impairment of endothelium-dependent vasodilation but also triggered a distinct pattern of endothelial biomarkers, compared to young mice. In particular, in aged mice, a distinct pattern of response to LPS was observed, as assessed by biomarkers of glycocalyx injury (SDC-1, ESM-1), endothelial permeability (Angpt-2), some biomarkers of endothelial hemostasis (sTM, TAFI, THBS-1), while LPS induced a similar response in aged and young mice in terms of classical hemostasis biomarkers (PAI-1, t-PA and vWF) and biomarkers of endothelial inflammation (sVCAM-1, sICAM-1, sE-sel, sP-sel). Importantly, aged mice displayed also impaired baseline NO-dependent endothelial function and alterations in number of endothelial biomarkers including those related to glycocalyx injury, endothelial permeability and endothelial hemostasis (higher SDC-1, Angpt-2, vWF, PAI-1, TAFI), suggesting that aging itself modulates the same pathways that are differentially affected by LPS in aged *vs*. young mice.

Accordingly, glycocalyx injury, regulation of endothelial permeability, and specific mechanisms of hemostasis may constitute the basis for specific-age-related biomarkers reflecting distinct response to LPS of endothelium in aged mice as compared to young mice. If so, panel of well-selected endothelial biomarkers (SDC-1, ESM-1 Angpt-2, sTM, TAFI, THBS-1) rather than classical biomarkers of hemostasis and of endothelial inflammation may prove useful to detect distinct profile of endothelial response to LPS.

One important aspect of the current study was that we used a relatively low dose of LPS (3 mg/kg) that did not induce higher mortality in aged mice. Even though a relatively low dose of LPS was used, some biomarkers of liver and of kidney injury in particular were higher in aged mice than in young mice following LPS, in line with our previous studies showing greater increase in kidney injury biomarkers as compared to liver injury biomarkers after LPS in mice [10, 59]. Despite only modest differences in plasma urea and ALT levels—and no significant changes in other liver (AST) or kidney (creatinine) injury biomarkers between aged and young mice following LPS exposure—we observed a distinct endothelial response profile in aged mice. This response was characterized not only by more pronounced impairment of NO-dependent vasodilation but also by more pronounced glycocalyx injury, impaired endothelial permeability, and altered hemostasis offering a novel insight into age-specific mechanisms of endothelial response to LPS.

Several previous studies suggested that glycocalyx injury contributed to organ injury in sepsis [60] and there was a number of studies analyzing glycocalyx biomarkers in sepsis. For example, SDC-1 or ESM-1 was reported to increase in patients with sepsis and their high plasma levels were associated with the severity and mortality in sepsis [61–63]. Interestingly, Sheng-Yuan Hsiao et al. assessed serial changes in biomarkers of endothelial dysfunction in patients with severe sepsis and finally classified ESM-1 as a good prognostic biomarker in clinical practice and treatment for severe sepsis [64], though other authors suggested ESM-1 should be used simultaneously with other endothelial specific biomarkers to predict the outcome of sepsis [65]. Our study adds another aspect to the possible value of glycocalyx biomarkers in predicting sepsis outcome underscoring that distinct pattern of glycocalyx biomarkers at basal state and in response to LPS in aged vs. young mice could represent a specific age-dependent pathomechanism contributing to the more pronounced vascular injury induced by LPS associated with aging.

Similarly, previous studies provided evidence that increased endothelial permeability contributed to the pathophysiology of vascular and organ injury in sepsis, which could be manifested by disturbances in angiopoietin/Tie2 axis [66] and the vascular endothelial growth factor (VEGF) pathway [67]. Parikh et al. showed that Angpt-2 released by activated endothelial cells increased in patients with sepsis [68], while Greco et al. presented that sFLT-1 levels were higher in patients with complicated sepsis [67]. In fact, both Angpt-2 and sFLT-1 were classified as the useful prognostic biomarkers for sepsis severity [67, 69–71].

Our study revealed that specific age-dependent pathomechanisms of more pronounced vascular injury associated with aging could be related to more intense changes in endothelial permeability with detected alterations in Angpt-2 in basal conditions and alterations in angiopoietin/Tie2 axis in response to LPS in aged vs. young mice. However, there was no distinct response related to VEGF pathway. Of note, the activity of angiopoietin/Tie2 axis was shown to be tightly linked to glycocalyx integrity [66, 72]. Therefore, these results seem compatible with more intense glycocalyx injury in aged mice. Taken together glycocalyx injury and alterations in endothelial permeability appeared as major aspects of endothelial function that displayed a distinct response to LPS in aged mice. These results would suggest that pharmacological targeting of glycocalyx and endothelial permeability could be of special therapeutic benefit in aged vasculature and treatment of enhanced LPS-induced vascular injury associated with aging [73, 74].

Interestingly, classical biomarkers of hemostasis (e.g., vWF, PAI-1, t-PA) did not respond differently to LPS in aged vs. young mice, while sTM, TAFI, and THBS-1 did show apparent differences. Moreover, aging was associated with higher level of classical and non-classical biomarkers of altered hemostasis (vWF, PAI-1 and TAFI) suggesting that age-dependent changes in endothelial-dependent regulation of hemostasis determine the distinct pattern of LPS-induced response in aged vs. young mice. In previous studies, sTM as well as TAFI were shown to predict sepsis outcome [75, 76], while THBS-1 was proposed as biomarker of myocardial injury in sepsis [77]. In particular, sTM [30, 66, 78–80] was mechanistically linked with severity of endothelial damage and thrombin generation [75], while TAFI was shown to play a major role in the inhibition of fibrinolysis [81, 82]. In turn, recombinant TM inhibited pulmonary endothelial glycocalyx injury and attenuated ventilator-induced lung injury in endotoxemia [83].

Although coagulation processes are tightly linked with inflammatory processes in sepsis [84, 85], the distinct pattern of sTM, TAFI, and THBS-1 response to LPS in aged mice vs. young mice was not associated with more pronounced endothelial inflammation (sVCAM-1, sICAM-1, sE-sel, sP-sel).

In several previous reports, the inflammatory response in aged mice was found to enhanced [86, 87], and organ damage was also found to be more severe than in young mice [10, 88]. Our results show also some differences in the dynamics of inflammation induced by relatively low dose of LPS, with only three inflammatory mediators (IL-1β, IL-2, and eotaxin) displaying consistently different response in aged vs. young mice. However, most of the other cytokines/chemokines show very modest age-dependent difference. These results suggest that the distinct profile of endothelial biomarker response represents a *sensitive readout* reflecting age-dependent difference in response to LPS in aged mice as compared to young mice that could not be explained by different levels of systemic inflammation in aged vs. young mice. 

It is well known that aging results in impaired bioavailability of NO [14, 89, 90]. Our results showed that aged mice displayed pre-existing, age-related endothelial dysfunction as well as more pronounced endothelial dysfunction after LPS as assessed in vivo by MRI using previously validated methodology [14, 42, 43, 48, 51, 91]. Accordingly, age-dependent deterioration of NO-dependent function, a distinct feature of aging vasculature [12–14] seemed to predispose to more severely impairment of NO-dependent function after LPS in aged mice. This finding stays in line with our recent report showing that age-dependent pre-existing endothelial dysfunction predisposed to rapid EndMT progression in response to breast cancer cells [50].

Taken together, our findings suggest that aging-specific changes in endothelial phenotype predispose to increased vulnerability of aging vasculature to LPS exemplified by more severe impairment of NO-dependent function, glycocalyx injury, increased endothelial permeability. and alterations in selected mechanisms of hemostasis, but not classical mechanisms of endothelial inflammation and hemostasis in aged mice vs. young mice.

An important conclusion of this study is that alterations in NO-dependent endothelial function that differentiate LPS response in aged mice vs. young mice are not consistently reflected by the classical panel of pro-inflammatory and pro-thrombotic blood biomarkers. Instead, our results highlight a disease-specific profile of endothelial response, the notion that appears consistent with our previous studies in mice. Indeed, in atherosclerosis, impaired NO-dependent function detected in vivo by MRI was related to the loss of glycocalyx integrity, increased endothelial permeability and elevation of typical biomarkers of endothelial inflammation and hemostasis [42]. In mice fed a high-fat diet, which also showed a severe impairment of NO-dependent function assessed in vivo, there was no corresponding elevation in classical systemic biomarkers of endothelial dysfunction [43]. Similarly, in humanized model of mild hyperlipidemia, we detected progressive age-dependent impairment of NO-dependent function with no changes in classical biomarkers of endothelial inflammation [51]. However, these biomarkers characterized endothelial dysfunction in a murine model of cancer metastasis [49, 50].

There are couple of limitations of this study. The main limitation is that there were relatively small groups of young and aged mice so we could have missed some differences if modest between groups. Still, we detected a distinct pattern of endothelial response in aged vs. young mice. The functional analysis performed by MRI was done only at two timepoints after LPS administration (2 h and 12 h after LPS), while five timepoints were chosen for biochemical analyses. However, the level of functional impairment of endothelial function 2 and 12 h after LPS was similar. We used a moderate dose of LPS that did not show unequivocally more severe organ injury and substantial difference in systemic inflammation in aged vs. young mice. However, such a dose of LPS enabled us to show the distinct profile of endothelial response in aged vs. young mice in this experimental settings. The targeted proteomics approach allowed us to measure plasma concentrations of selected biomarkers reflecting specific aspects of endothelial function (e.g., glycocalyx damage, endothelial permeability, endothelial inflammation), but we did not investigate the function of these proteins in the pathogenesis of endotoxemia-induced endothelial dysfunction, nor were we able to indicate the organ-specific localization of endothelial changes reflected by the systemic levels of selected biomarkers.

Despite this limitations, results presented in the current report demonstrate that the age-dependent deterioration of endothelial function results in a specific endotype of endothelial response to LPS. Accordingly, our report identifies the specific pattern of biomarkers (SDC-1, ESM-1, Angpt-2, sTM, TAFI, THBS-1) that could be more specific for detecting age-specific response of endothelium to LPS than classical biomarkers of endothelial inflammation and hemostasis, which are commonly used to assess endothelial status in many studies. Further studies are needed to translate these findings to the clinical context of older patients and to evaluate their increased susceptibility to sepsis-related vascular injury based on the identified biomarkers.

## Supplementary Information

Below is the link to the electronic supplementary material.

ESM 1 Body weight and weight of selected organs in young and aged C57BL/6 mice with induced endotoxemia by administration of LPS (3 mg/kg). Measurement of body weight and weight of selected organs in young and aged C57BL/6 mice was performed in each experimental group which consisted of 10 individuals. The results are presented as means (–) ± SEM. *, **, *** indicate statistically significant difference between young mice and aged mice at the same timepoint with *p*<0.05, *p*<0.01, and *p*<0.001, respectively. (PNG 456 KB)

High Resolution Image (TIF 3.89 MB)

ESM 2 Selected parameters characterizing the degree of organ damage and determining the functional state of the body measured in young, 3-month-old mice after single intraperitoneal administration of LPS at three different doses: 3mg/kg, 5mg/kg and 10mg/kg. Selected parameters characterizing the degree of organ damage and determining the functional state of the body were measured in one control group of mice (*n*=9-21) and in 4 experimental groups of mice (1 h, 3 h, 12 h, 24 h after LPS administration) for two doses: 3 mg/kg (*n*=6-8), 5 mg/kg (*n*=6-7) and in 5 experimental groups of mice (1h, 2 h, 3 h, 6 h, 12 h after LPS administration) for one dose: 10 mg/kg (*n*=6-20) in young, 3-month-old mice. The results are presented as means (–) ± SEM. ** indicate statistically significant difference between young mice and aged mice at the same timepoint with *p*<0.01. #, ##,### indicate statistically significant difference between young control mice and other studied groups of young animals after administration of LPS at a dose of 3 mg/kg with *p*<0.05, *p*<0.01, and *p*<0.001, respectively. #, ##, ### indicate statistically significant difference between young control mice and other studied groups of young animals after administration of LPS at a dose of 5 mg/kg with *p*<0.05, *p*<0.01, and *p*<0.001, respectively. #, ##, ### indicate statistically significant difference between young control mice and other studied groups of young animals after administration of LPS at a dose of 10 mg/kg with *p*<0.05, *p*<0.01, and *p*<0.001, respectively. Abbreviations: alanine transaminase (ALT), aspartate transaminase (AST), glutathione (GSH), nitrate (NO_3_^-^), platelets (PLT), ratio glutathione/glutathione disulfide (ratio GSH/GSSG), serum amyloid A (SAA), white blood cells (WBC). (PNG 954 KB)

High Resolution Image (TIF 5.52 MB)

ESM 3 Changes in the response of selected proteins specific for endothelial dysfunction measured in plasma of young C57BL/6 mice depending on the used LPS dose. Panel of selected plasma biomarkers specific for glycocalyx disruption, endothelial inflammation, endothelial permeability and hemostasis was measured in one control group of mice (*n*=9-16) and in 4 experimental groups of mice (1 h, 3 h, 12 h, 24 h after LPS administration) for two doses: 3 mg/kg (*n*=6-8), 5 mg/kg (*n*=7) and in 5 experimental groups of mice (1 h, 2 h, 3 h, 6 h, 12 h after LPS administration) for one dose: 10 mg/kg (*n*=5-16) in young, 3-month-old mice. The results are presented as means (–) ± SEM. *, *** indicate statistically significant difference between young mice and aged mice at the same timepoint with *p*<0.05 and *p*<0.001, respectively. #, ##, ### indicate statistically significant difference between young control mice and other studied groups of young animals after administration of LPS at a dose of 3 mg/kg with *p*<0.05, *p*<0.01, and *p*<0.001, respectively. #, ##, ### indicate statistically significant difference between young control mice and other studied groups of young animals after administration of LPS at a dose of 5 mg/kg with *p*<0.05, *p*<0.01, and *p*<0.001, respectively. ### indicate statistically significant difference between young control mice and other studied groups of young animals after administration of LPS at a dose of 10 mg/kg with *p*<0.001. Abbreviations: angiopoietin 1 (Angpt-1), syndecan-1 (SDC-1), the soluble form of E-selectin (sE-sel), the soluble form of P-selectin (sP-sel), thrombin activatable fibrinolysis inhibitor (TAFI), thrombospondin 1 (THBS-1). (PNG 491 KB)

High Resolution Image (TIF 3.27 MB)

ESM 4 Principal Component Analysis (PCA) plot showing the multivariate variation among 20 biomarkers specific for endothelial dysfunction in terms of age of mice and time after *i.p.* administration of LPS. Presented data are shown in two ways as (A) Score Plot and (B) Loading Plot. Size of circles indicate on the age of mice taken into experiment. Colored symbols correspond to the six experimental groups of mice. The first two principal axes explained 45.81% of the variance. (PNG 479 KB)

High Resolution Image (TIF 3.45 MB)

ESM 5 Principal Component Analysis (PCA) plot showing the multivariate variation among 23 cytokines/chemokines in terms of age of mice and time after *i.p.* administration of LPS. Presented data are shown in two ways as (A) Score Plot and (B) Loading Plot. Size of circles indicate on the age of mice taken into experiment. Colored symbols correspond to the six experimental groups of mice. The first two principal axes explained almost 75% of the variance. (TIF 3.35 MB)

High Resolution Image (PNG 479 KB)

ESM 6 Changes in the concentration of selected proteins measured in plasma by western blots. Legend: control- group of 3-month-old control mice (*n* = 8), LPS– group of 3-month-old mice 12 h after administration of LPS at a dose of 3 mg/kg (*n* = 6-8); TPS– total protein staining (loading control). Abbreviations: annexin A5 (ANXA5), the soluble form of transmembrane tyrosine-protein kinase receptor for Angpt-1, Angpt-2 and Angpt-4 (sTie-2), the soluble form of thrombomodulin (sTM). (PNG 391 KB)

High Resolution Image (TIF 4.59 MB)

ESM 7 (DOCX 44.7 KB)

ESM 8 (DOCX 23.5 KB)

ESM 9 (DOCX 66.5 KB)

ESM 10 (DOCX 41.7 KB)

ESM 11 (DOCX 55.4 KB)

ESM 12 (DOCX 47.7 KB)

ESM 13 (DOCX 74.1 KB)

## Data Availability

The data, analytic methods, and study materials will be made available on reasonable request to other researchers for the purpose of reproducing the results or replicating the procedure. The data from targeted proteomic analysis and cytokines/chemokines analysis were deposited to the RODBUK Cracow Open Research Data Repository (10.57903/UJ/MQOMB8).
