# Supplementary figures and images for: Distinct patterns of endothelial response to endotoxin in aged mice as compared to young mice
Source: GeroScience. 2025 Nov 26;48(2):1981–99. doi: 10.1007/s11357-025-01838-9 (PMC12972439; doi:10.1007/s11357-025-01838-9)

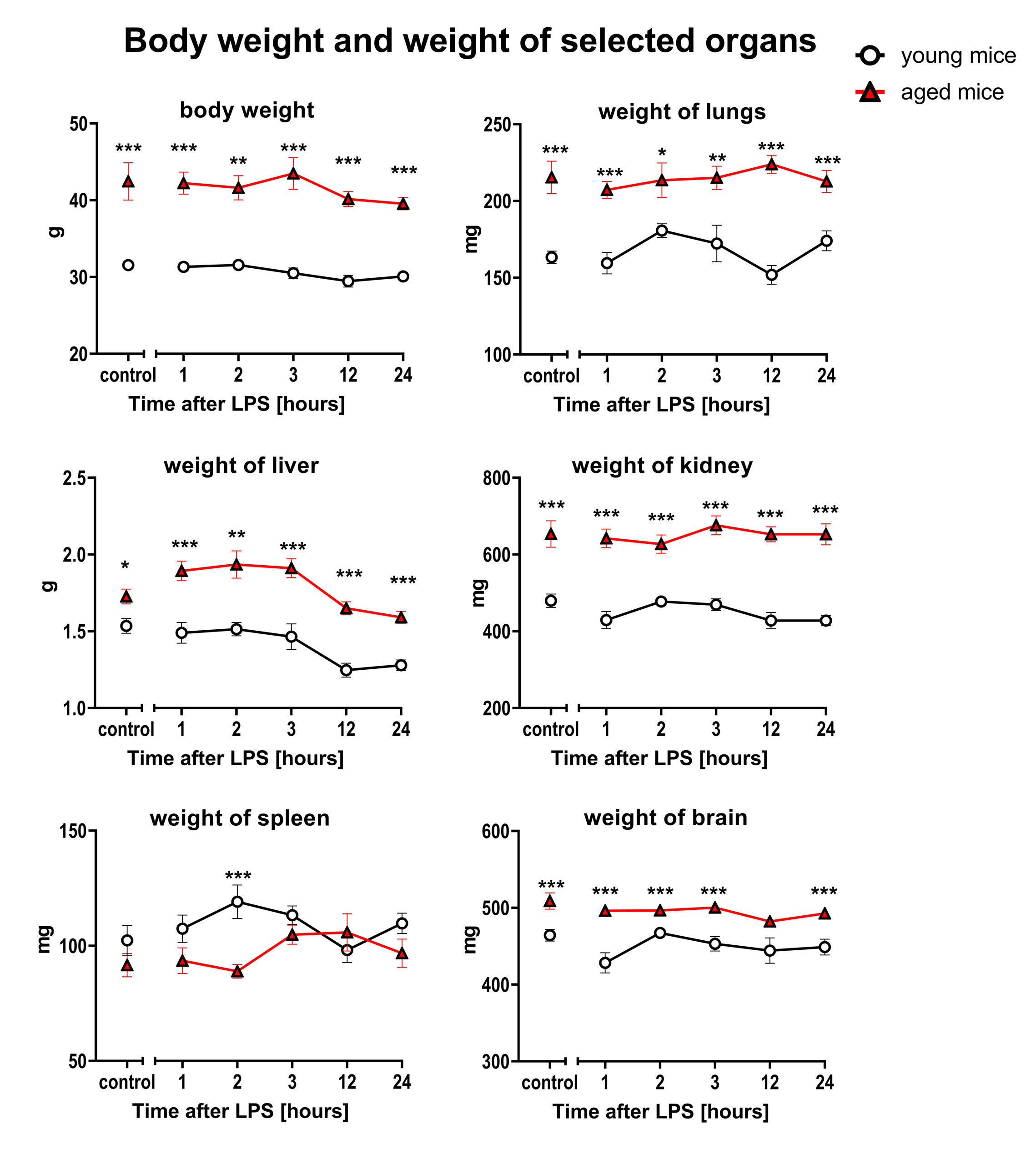

Supplement: Supplementary file 1 — Body weight and weight of selected organs in young and aged C57BL/6 mice with induced endotoxemia by administration of LPS (3 mg/kg). Measurement of body weight and weight of selected organs in young and aged C57BL/6 mice was performed in each experimental group which consisted of 10 individuals. The results are presented as means (–) ± SEM. *, **, *** indicate statistically significant difference between young mice and aged mice at the same timepoint with p<0.05, p<0.01, and p<0.001, respectively. (PNG 456 KB) [file 11357_2025_1838_Fig10_ESM.png]

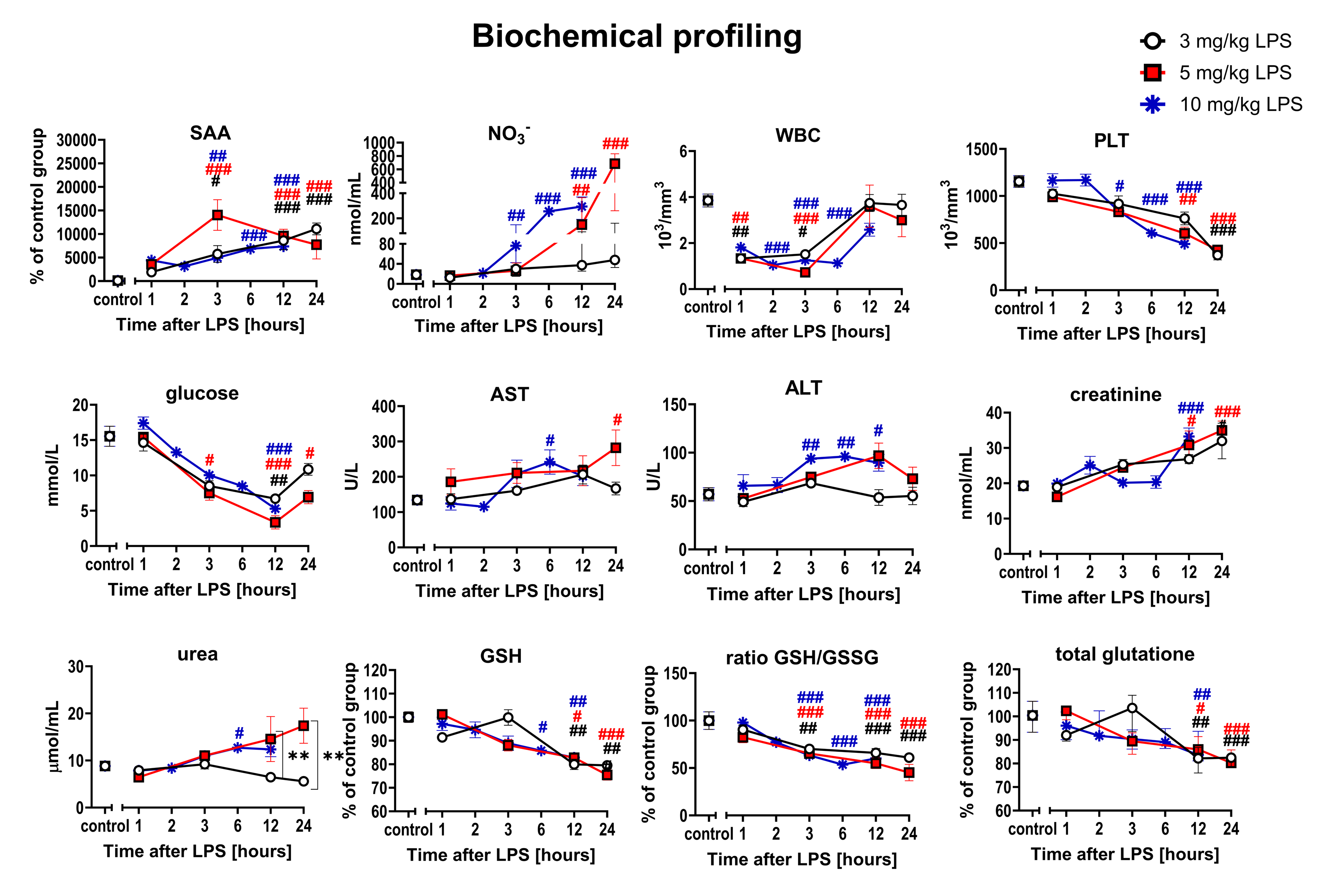

Supplement: Supplementary file 3 — Selected parameters characterizing the degree of organ damage and determining the functional state of the body measured in young, 3-month-old mice after single intraperitoneal administration of LPS at three different doses: 3mg/kg, 5mg/kg and 10mg/kg. Selected parameters characterizing the degree of organ damage and determining the functional state of the body were measured in one control group of mice (n=9-21) and in 4 experimental groups of mice (1 h, 3 h, 12 h, 24 h after LPS administration) for two doses: 3 mg/kg (n=6-8), 5 mg/kg (n=6-7) and in 5 experimental groups of mice (1h, 2 h, 3 h, 6 h, 12 h after LPS administration) for one dose: 10 mg/kg (n=6-20) in young, 3-month-old mice. The results are presented as means (–) ± SEM. ** indicate statistically significant difference between young mice and aged mice at the same timepoint with p<0.01. #, ##,### indicate statistically significant difference between young control mice and other studied groups of young animals after administration of LPS at a dose of 3 mg/kg with p<0.05, p<0.01, and p<0.001, respectively. #, ##, ### indicate statistically significant difference between young control mice and other studied groups of young animals after administration of LPS at a dose of 5 mg/kg with p<0.05, p<0.01, and p<0.001, respectively. #, ##, ### indicate statistically significant difference between young control mice and other studied groups of young animals after administration of LPS at a dose of 10 mg/kg with p<0.05, p<0.01, and p<0.001, respectively. Abbreviations: alanine transaminase (ALT), aspartate transaminase (AST), glutathione (GSH), nitrate (NO3-), platelets (PLT), ratio glutathione/glutathione disulfide (ratio GSH/GSSG), serum amyloid A (SAA), white blood cells (WBC). (PNG 954 KB) [file 11357_2025_1838_Fig11_ESM.png]

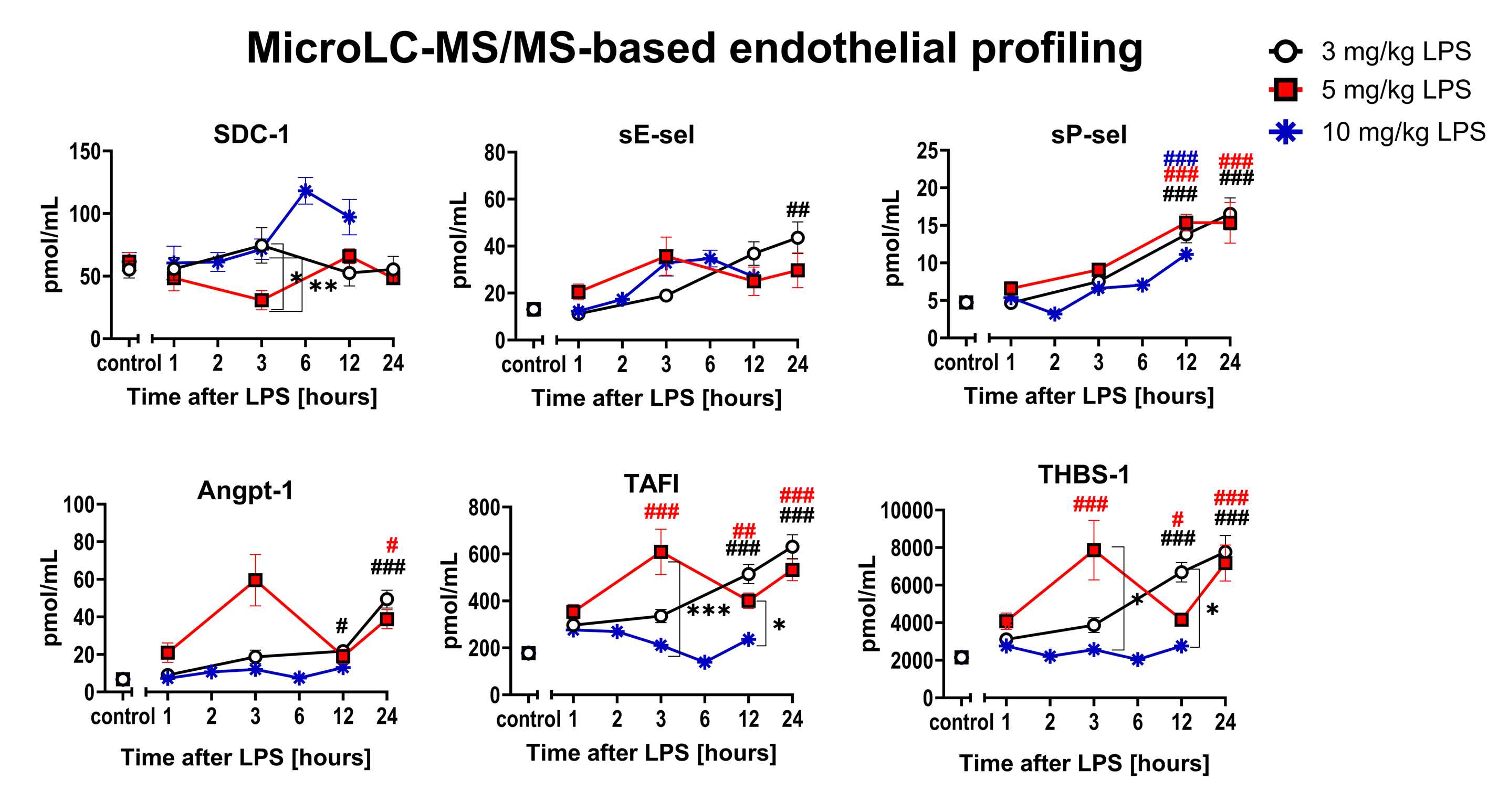

Supplement: Supplementary file 5 — Changes in the response of selected proteins specific for endothelial dysfunction measured in plasma of young C57BL/6 mice depending on the used LPS dose. Panel of selected plasma biomarkers specific for glycocalyx disruption, endothelial inflammation, endothelial permeability and hemostasis was measured in one control group of mice (n=9-16) and in 4 experimental groups of mice (1 h, 3 h, 12 h, 24 h after LPS administration) for two doses: 3 mg/kg (n=6-8), 5 mg/kg (n=7) and in 5 experimental groups of mice (1 h, 2 h, 3 h, 6 h, 12 h after LPS administration) for one dose: 10 mg/kg (n=5-16) in young, 3-month-old mice. The results are presented as means (–) ± SEM. *, *** indicate statistically significant difference between young mice and aged mice at the same timepoint with p<0.05 and p<0.001, respectively. #, ##, ### indicate statistically significant difference between young control mice and other studied groups of young animals after administration of LPS at a dose of 3 mg/kg with p<0.05, p<0.01, and p<0.001, respectively. #, ##, ### indicate statistically significant difference between young control mice and other studied groups of young animals after administration of LPS at a dose of 5 mg/kg with p<0.05, p<0.01, and p<0.001, respectively. ### indicate statistically significant difference between young control mice and other studied groups of young animals after administration of LPS at a dose of 10 mg/kg with p<0.001. Abbreviations: angiopoietin 1 (Angpt-1), syndecan-1 (SDC-1), the soluble form of E-selectin (sE-sel), the soluble form of P-selectin (sP-sel), thrombin activatable fibrinolysis inhibitor (TAFI), thrombospondin 1 (THBS-1). (PNG 491 KB) [file 11357_2025_1838_Fig12_ESM.png]

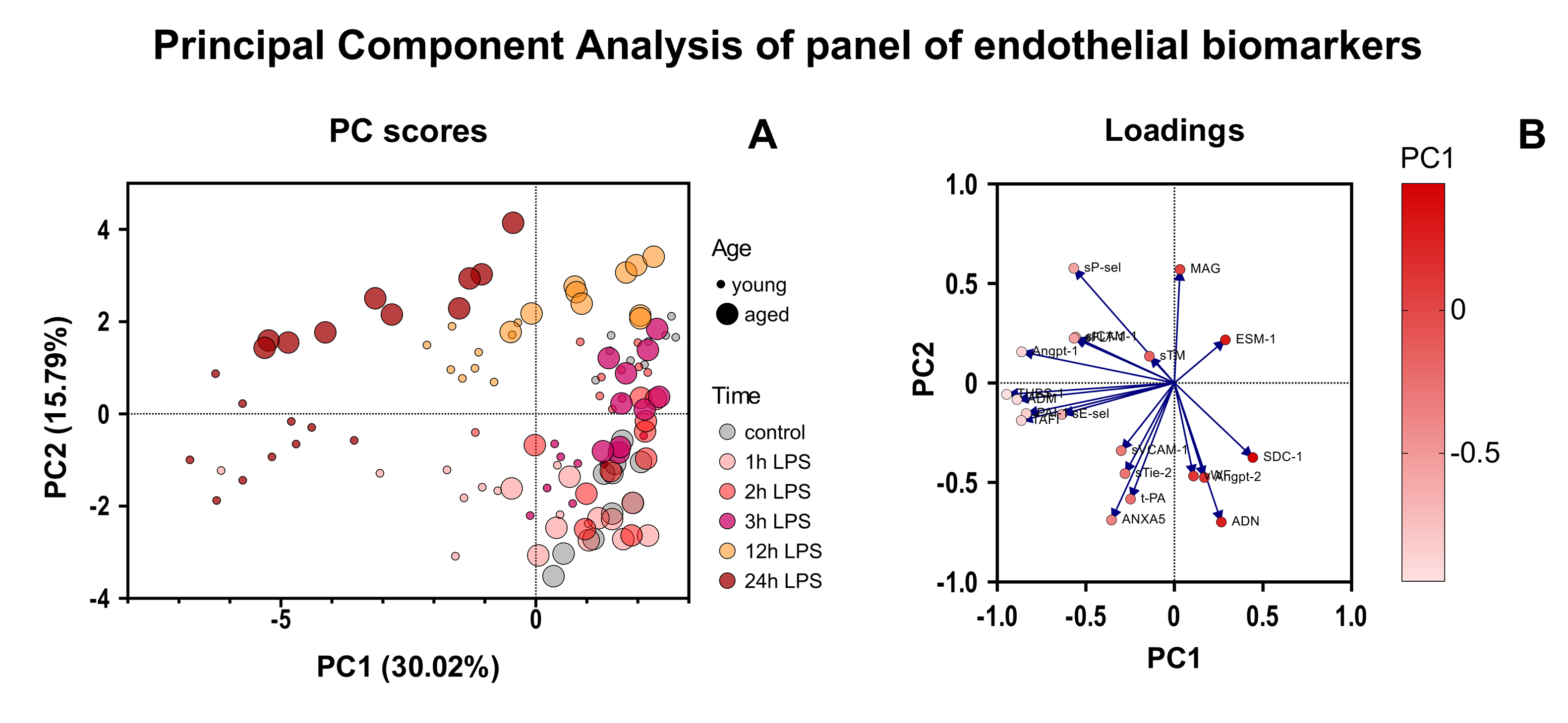

Supplement: Supplementary file 7 — Principal Component Analysis (PCA) plot showing the multivariate variation among 20 biomarkers specific for endothelial dysfunction in terms of age of mice and time after i.p. administration of LPS. Presented data are shown in two ways as (A) Score Plot and (B) Loading Plot. Size of circles indicate on the age of mice taken into experiment. Colored symbols correspond to the six experimental groups of mice. The first two principal axes explained 45.81% of the variance. (PNG 479 KB) [file 11357_2025_1838_Fig13_ESM.png]

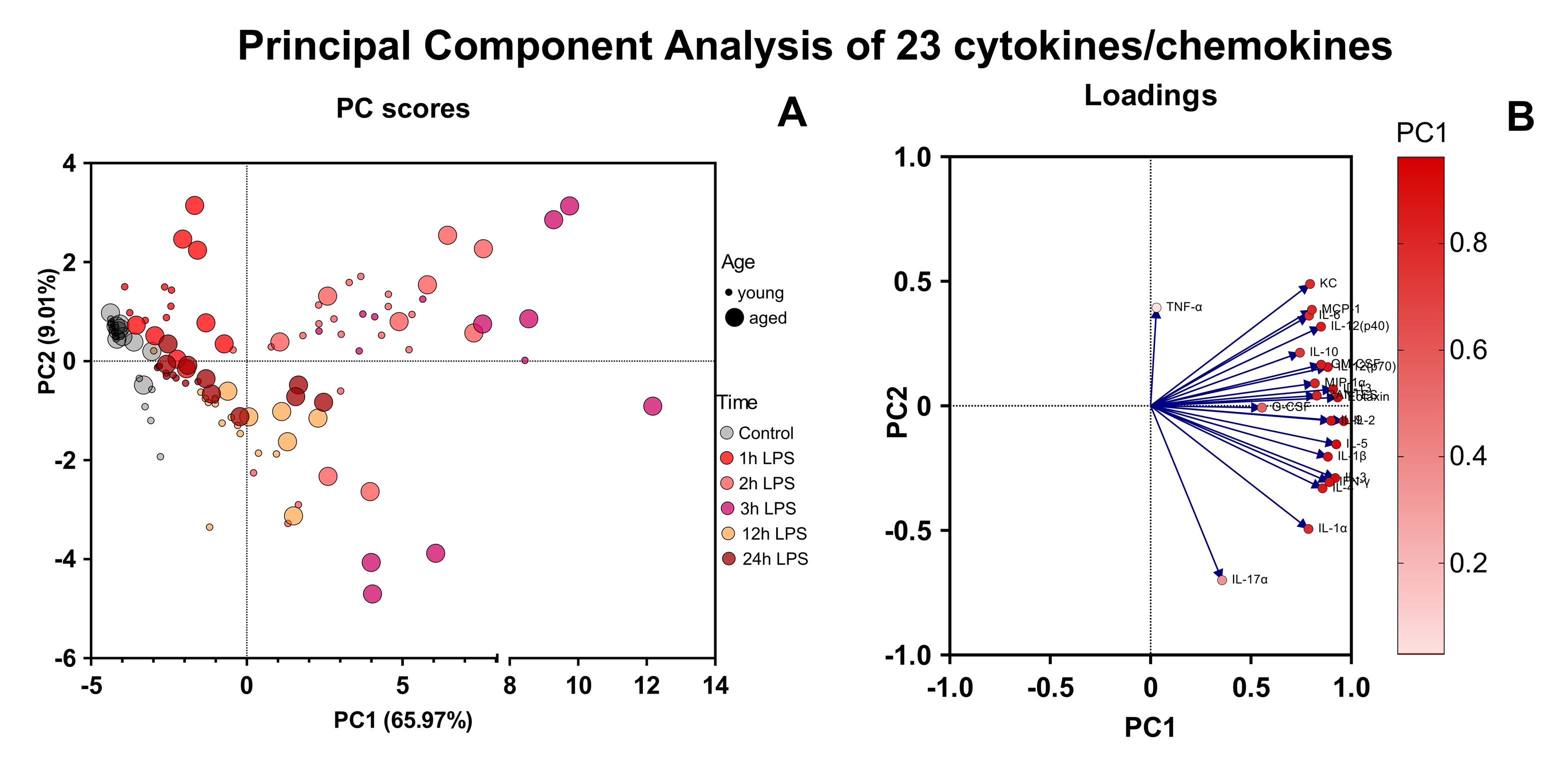

Supplement: Supplementary file 9 — Principal Component Analysis (PCA) plot showing the multivariate variation among 23 cytokines/chemokines in terms of age of mice and time after i.p. administration of LPS. Presented data are shown in two ways as (A) Score Plot and (B) Loading Plot. Size of circles indicate on the age of mice taken into experiment. Colored symbols correspond to the six experimental groups of mice. The first two principal axes explained almost 75% of the variance. (TIF 3.35 MB) [file 11357_2025_1838_Fig14_ESM.png]

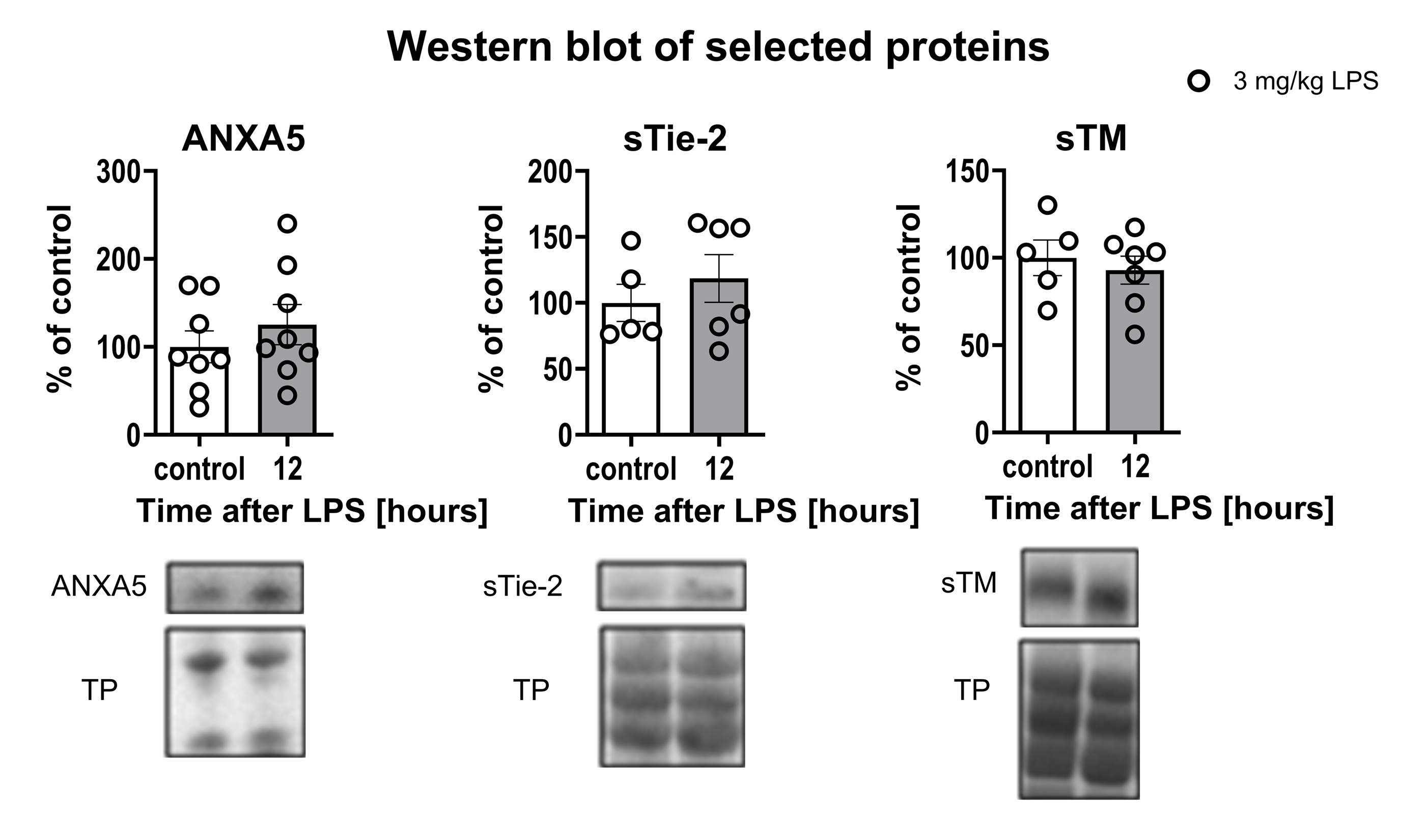

Supplement: Supplementary file 11 — Changes in the concentration of selected proteins measured in plasma by western blots. Legend: control- group of 3-month-old control mice (n = 8), LPS– group of 3-month-old mice 12 h after administration of LPS at a dose of 3 mg/kg (n = 6-8); TPS– total protein staining (loading control). Abbreviations: annexin A5 (ANXA5), the soluble form of transmembrane tyrosine-protein kinase receptor for Angpt-1, Angpt-2 and Angpt-4 (sTie-2), the soluble form of thrombomodulin (sTM). (PNG 391 KB) [file 11357_2025_1838_Fig15_ESM.png]
