# Supplementary material for: Distinct patterns of endothelial response to endotoxin in aged mice as compared to young mice
Source: GeroScience. 2025 Nov 26;48(2):1981–99. doi: 10.1007/s11357-025-01838-9 (PMC12972439; doi:10.1007/s11357-025-01838-9)
Supplement: Supplementary file 13 — (DOCX 44.7 KB) [file 11357_2025_1838_MOESM7_ESM.docx]

**Suppl. Table 1.1** Specific peptide sequences for Angpt-1, sTie-2, ANXA5, sP-sel, sTM, MAG, THBS-1 and TAFI and their SISs used in the quantitative analysis by microLC/MS-MRM method

| **Protein** | **Uniprot accession number** | **Target endogenous peptide sequence** | **Peptide MW (Da)** | **1 mg/mL of stock solutions of analytes expressed in µmol/mL** | **Target SIS peptide sequence** | **SIS Peptide MW (Da)** | **1 mg/mL of stock solutions of SISs expressed in µmol/mL** |
| --- | --- | --- | --- | --- | --- | --- | --- |
| Angpt-1 | O08538 | DAPHVEPDFSSQ**K** | 1456.51 | 0.69 | DAPHVEPDFSSQ**(K*)** | 1464.46 | 0.68 |
|  |  | LEIQLLENSLSTY**K** | 1650.87 | 0.60 | LEIQLLENSLSTY**(K*)** | 1658.81 | 0.60 |
|  |  | GHTGTAG**K** | 727.77 | 1.37 | GHTGTAG**(K*)** | 735.71 | 1.36 |
| sTie-2 | Q02858 | YIGGNLFTSAFT**R** | 1446.60 | 0.69 | YIGGNLFTSAFT**(R*)** | 1456.53 | 0.69 |
|  |  | EEDAVIY**K** | 966.04 | 1.04 | EEDAVIY**(K*)** | 973.99 | 1.03 |
|  |  | FSVAIFTVN**R** | 1153.33 | 0.87 | FSVAIFTVN**(R*)** | 1163.26 | 0.86 |
| ANXA5 | P48036 | TPEELSAI**K** | 987.1 | 1.01 | TPEELSAI**(K*)** | 995.05 | 1.00 |
|  |  | GAGTDDHTLI**R** | 1155.22 | 0.87 | GAGTDDHTLI**(R*)** | 1165.15 | 0.86 |
|  |  | FITIFGT**R** | 954.12 | 1.05 | FITIFGT**(R*)** | 964.05 | 1.04 |
| sP-sel | Q01102 | GITSLPAPAV**R** | 1081.27 | 0.92 | GITSLPAPAV**(R*)** | 1091.20 | 0.92 |
|  |  | SNSAPG**K** | 659.69 | 1.52 | SNSAPG**(K*)** | 667.63 | 1.50 |
| sTM | P15306 | EVVLQHV**R** | 979.13 | 1.02 | EVVLQHV**(R*)** | 989.06 | 1.01 |
|  |  | LQGHLMTV**R** | 1054.27 | 0.95 | LQGHLMTV**(R*)** | 1064.20 | 0.94 |
|  |  | GHLMTV**R** | 812.98 | 1.23 | GHLMTV**(R*)** | 822.91 | 1.22 |
| MAG | P20917 | TQVVHESFQG**R** | 1287.38 | 0.78 | TQVVHESFQG**(R*)** | 1297.31 | 0.77 |
|  |  | LLGDLGL**R** | 856.02 | 1.17 | LLGDLGL**(R*)** | 865.95 | 1.15 |
|  |  | EFVYSE**R** | 928.98 | 1.08 | EFVYSE**(R*)** | 938.91 | 1.07 |
| THBS-1 | P35441 | SITLFVQED**R** | 1207.33 | 0.83 | SITLFVQED**(R*)** | 1217.26 | 0.82 |
|  |  | LVPNPDQ**K** | 910.03 | 1.10 | LVPNPDQ**(K*)** | 917.97 | 1.09 |
|  |  | AQGYSGLSV**K** | 1009.11 | 1.00 | AQGYSGLSV**(K*)** | 1017.06 | 0.98 |
| TAFI | Q9JHH6 | YGFLLPE**R** | 994.14 | 1.01 | YGFLLPE**(R*)** | 1004.07 | 1.00 |
|  |  | IYIGSSFE**K** | 1043.17 | 0.96 | IYIGSSFE**(K*)** | 1051.11 | 0.95 |
|  |  | YSFTIEL**R** | 1028.16 | 0.97 | YSFTIEL**(R*)** | 1038.09 | 0.96 |
