# Supplementary material for: Distinct patterns of endothelial response to endotoxin in aged mice as compared to young mice
Source: GeroScience. 2025 Nov 26;48(2):1981–99. doi: 10.1007/s11357-025-01838-9 (PMC12972439; doi:10.1007/s11357-025-01838-9)
Supplement: Supplementary file 14 — (DOCX 23.5 KB) [file 11357_2025_1838_MOESM8_ESM.docx]

|  | **Young, 3-month-old mice** | | | | | | **Aged, 18-month-old mice** | | | | | |
| --- | --- | --- | --- | --- | --- | --- | --- | --- | --- | --- | --- | --- |
| **Parameter** | **control** | **1h LPS** | **2h LPS** | **3h LPS** | **12h LPS** | **24h LPS** | **control** | **1h LPS** | **2h LPS** | **3h LPS** | **12h LPS** | **24h LPS** |
| **NO_3_^-^**  **[nmol/mL]** | 12.88±2.21  n=9 | 7.13±1.68  n=8 | 22.53±2.49  n=9 | 25.21±5.06  n=8 | 126.5±33.4  n=8 | 213.4±57.5  n=10 | 24.30±2.28  ******  n=9 | 21.71±2.01  *******  n=10 | 19.67±2.22  n=10 | 19.92±2.45  n=7 | 155.1±16.6  n=9 | 174.1±47.1  n=10 |
| **WBC [10^3^/mm^3^]** | 4.50±0.50  n=9 | 1.51±0.33  n=10 | 0.92±0.16  n=9 | 1.41±0.25  n=8 | 1.74±0.20  n=8 | 1.50±0.20  n=10 | 5.20±0.40  n=10 | 2.13±0.31  n=10 | 0.93±0.12  n=9 | 0.92±0.09  n=10 | 1.59±0.29  n=10 | 1.60±0.22  n=10 |
| **PLT [10^3^/mm^3^]** | 884.7±37.1  n=9 | 850.8±42.8  n=10 | 592.6±57.3  n=10 | 481.9±50.7  n=8 | 473.3±38.1  n=8 | 312.3±30.4  n=9 | 1312±59  *******  n=10 | 1105±44  *******  n=10 | 927.9±47.3  *******  n=10 | 1041±38  *******  n=10 | 639.6±114.6  n=10 | 514.3±56.6  ******  n=9 |
| **RBC [10^6^/mm^3^]** | 9.19±0.26  n=9 | 9.26±0.30  n=10 | 10.20±0.23  n=10 | 10.38±0.21  n=8 | 10.26±0.17  n=9 | 10.14± 0.10  n=10 | 9.17±0.22  n=10 | 9.92±0.35  n=10 | 10.66±0.30  n=10 | 10.61±0.20  n=10 | 9.92±0.33  n=10 | 8.96±0.49  *****  n=10 |
| **HGB [g/dL]** | 13.76±0.37  n=9 | 13.63±0.43  n=10 | 14.96±0.32  n=10 | 15.05±0.29  n=8 | 15.11±0.22  n=8 | 14.90±0.15  n=10 | 13.41±0.26  n=10 | 14.22±0.41  n=10 | 14.99±0.38  n=10 | 15.22±0.30  n=10 | 13.95±0.40  *****  n=10 | 12.87±0.53  ******  n=10 |
| **MCV [µm^3^]** | 48.11±0.20  n=9 | 47.20±0.25  n=10 | 47.70±0.21  n=10 | 47.88±0.40  n=8 | 47.25±0.41  n=8 | 46.70±0.26  n=10 | 48.15±0.35  n=10 | 47.75±0.40  n=10 | 47.30±0.34  n=10 | 47.50±0.38  n=10 | 45.90±0.19  ******  n=10 | 46.90±0.80  n=10 |
| **HCT**  **[%]** | 44.22±1.29  n=9 | 43.72±1.51  n=10 | 48.58±1.08  n=10 | 49.66±1.04  n=8 | 48.39±0.81  n=8 | 47.38±0.53  n=10 | 43.99±0.92  n=10 | 47.33±1.58  n=10 | 50.13±1.65  n=9 | 50.38±1.07  n=10 | 45.60±1.55  n=10 | 41.69±1.85  *****  n=10 |
| **MCH**  **[pg]** | 14.96±0.04  n=9 | 14.72±0.06  n=10 | 14.67±0.07  n=10 | 14.50±0.07  n=8 | 14.74±0.11  n=8 | 14.70±0.07  n=10 | 14.64±0.14  *****  n=10 | 14.37±0.12  *****  n=10 | 14.09±0.10  *******  n=10 | 14.38±0.15  n=10 | 14.01±0.10  *******  n=10 | 14.50±0.28  n=10 |
| **MCHC [g/dL]** | 31.12±0.13  n=9 | 31.18±0.12  n=10 | 30.81±0.07  n=10 | 30.34±0.08  n=8 | 31.24±0.10  n=8 | 31.48±0.15  n=10 | 30.46±0.08  *******  n=10 | 30.07±0.14  *******  n=10 | 29.81±0.15  *******  n=10 | 30.23±0.12  n=10 | 30.44±0.15  *******  n=10 | 30.93±0.17  *****  n=10 |
| **glucose**  **[mmol/L]** | 15.42±1.65  n=9 | 11.49±2.57  n=10 | 10.54±2.52  n=10 | 8.68±2.45  n=6 | 6.06±0.57  n=7 | 7.59±1.58  n=9 | 16.76±3.88  n=10 | 16.22±3.76  ******  n=10 | 15.46±2.80  *******  n=9 | 12.35±2.40  *****  n=9 | 5.82±2.46  n=8 | 5.66±1.74  *****  n=9 |

***Suppl. Table 1. Blood count, nitrate and glucose plasma levels along the development of endotoxemia induced by LPS (3 mg/kg) in young and aged C57BL/6 mice.***

The results are presented as means (–) ± SEM.

*, **, *** indicate statistically significant difference between young mice and aged mice at the same timepoint with p<0.05, p<0.01, and p<0.001, respectively.

**Abbreviations:** hematocrit (HCT), hemoglobin (HGB), mean corpuscular hemoglobin (MCH), mean corpuscular hemoglobin concentration (MCHC), mean cell volume (MCV), nitrate (NO_3_^-^), platelets (PLT), count of red blood cells (RBC), white blood cells (WBC).
