# Supplementary material for: Distinct patterns of endothelial response to endotoxin in aged mice as compared to young mice
Source: GeroScience. 2025 Nov 26;48(2):1981–99. doi: 10.1007/s11357-025-01838-9 (PMC12972439; doi:10.1007/s11357-025-01838-9)
Supplement: Supplementary file 15 — (DOCX 66.5 KB) [file 11357_2025_1838_MOESM9_ESM.docx]

**Suppl. Table 2.1** Optimized MRM parameters for the panel of 22 endogenously generated peptides specific for Angpt-1, sTie-2, ANXA5, sP-sel, sTM, MAG, THBS-1 and TAFI and their 22 stable isotope-labelled internal standards targeting these 8 proteins

| **Protein**  **MW (Da)** | **Uniprot accession number** | **Specific peptide sequence** | **Peptide**  **MW (Da)** | **MRM transition^a^** | | **Product ion** | **DP (V)** | **CE (V)** |
| --- | --- | --- | --- | --- | --- | --- | --- | --- |
|  |  |  |  | **Q1** | **Q3** |  |  |  |
| Angpt-1  ( 57 519) | O08538 | **DAPHVEPDFSSQK** | 1456.51 | **486.4** | **449.2** | **y_4_** | **45** | **18** |
|  |  |  |  | 486.4 | 635.9 | y_11_^2+^ | 45 | 16 |
|  |  | LEIQLLENSLSTYK | 1650.87 | 826.1 | 243.2 | b_2_ | 80 | 42 |
|  |  |  |  | 826.1 | 1054.5 | y_9_ | 80 | 33 |
|  |  | GHTGTAGK | 727.77 | 364.8 | 195.1 | b_2_ | 85 | 24 |
|  |  |  |  | 364.8 | 433.3 | y_5_ | 85 | 20 |
| sTie-2  ( 125 701) | Q02858 | YIGGNLFTSAFTR | 1446.60 | 724.1 | 1170.7 | y_11_ | 60 | 33 |
|  |  |  |  | 724.1 | 829.4 | y_7_ | 60 | 33 |
|  |  | **EEDAVIYK** | 966.04 | **483.9** | **423.4** | **y_3_** | **75** | **18** |
|  |  |  |  | 483.9 | 310.2 | y_2_ | 75 | 21 |
|  |  | FSVAIFTVNR | 1153.33 | 577.6 | 636.4 | y_5_ | 80 | 23 |
|  |  |  |  | 577.6 | 820.5 | y_7_ | 80 | 25 |
| ANXA5  ( 35 752) | P48036 | **TPEELSAIK** | 987.1 | **494.5** | **789.4** | **y_9_** | **70** | **23** |
|  |  |  |  | 494.5 | 443.9 | y_8_^2+^ | 70 | 25 |
|  |  | GAGTDDHTLIR | 1155.22 | 386.2 | 514.4 | y_9_^2+^ | 45 | 14 |
|  |  |  |  | 386.2 | 502.4 | y_4_ | 45 | 23 |
|  |  | FITIFGTR | 954.12 | 477.9 | 694.4 | y_6_ | 65 | 21 |
|  |  |  |  | 477.9 | 480.4 | y_4_ | 65 | 21 |
| sP-sel  ( 83 099) | Q01102 | **GITSLPAPAVR** | 1081.27 | **541.5** | **610.4** | **y_6_** | **45** | **21** |
|  |  |  |  | 541.5 | 472.3 | b_5_ | 45 | 16 |
|  |  | SNSAPGK | 659.69 | 331.3 | 301.3 | y_3_ | 45 | 18 |
|  |  |  |  | 331.3 | 459.3 | y_5_ | 45 | 14 |
| sTM  ( 61 868) | P15306 | EVVLQHV**R** | 979.13 | 327.2 | 411.5 | y_3_ | 60 | 19 |
|  |  |  |  | 327.2 | 321.3 | z_8_^3+^ | 60 | 8 |
|  |  | **LQGHLMTVR** | 1054.27 | **352.4** | **462.4** | **z_8_^2+^** | **60** | **16** |
|  |  |  |  | 352.4 | 506.2 | y_4_ | 60 | 21 |
| MAG  (69 388) | P20917 | TQVVHESFQG**R** | 1287.38 | 430.1 | 230.1 | b_2_ | 80 | 19 |
|  |  |  |  | 430.1 | 529.8 | y_9_^2+^ | 80 | 17 |
|  |  | **LLGDLGLR** | 856.02 | **429.0** | **199.3** | **a_2_** | **60** | **22** |
|  |  |  |  | 429.0 | 227.1 | b_2_ | 60 | 17 |
|  |  | EFVYSE**R** | 928.98 | 465.5 | 554.4 | y_4_ | 40 | 20 |
|  |  |  |  | 465.5 | 249.3 | a_2_ | 40 | 28 |
| THBS-1  ( 129 647) | P35441 | SITLFVQEDR | 1207.33 | 604.5 | 793.4 | y_6_ | 75 | 26 |
|  |  |  |  | 604.5 | 201.2 | b_2_ | 75 | 23 |
|  |  | LVPNPDQK | 910.03 | 455.9 | 349.8 | y_6_^2+^ | 50 | 16 |
|  |  |  |  | 455.9 | 487.4 | y_4_ | 50 | 25 |
|  |  | **AQGYSGLSVK** | 1009.11 | **505.4** | **590.4** | **y_6_** | **65** | **22** |
|  |  |  |  | 505.4 | 810.4 | y_8_ | 65 | 21 |
| TAFI  ( 48 871) | Q9JHH6 | YGFLLPER | 994.14 | 497.9 | 401.2 | y_3_ | 80 | 21 |
|  |  |  |  | 497.9 | 514.4 | y_4_ | 80 | 21 |
|  |  | IYIGSSFEK | 1043.17 | 522.5 | 767.4 | y_7_ | 75 | 21 |
|  |  |  |  | 522.5 | 277.2 | b_2_ | 75 | 22 |
|  |  | **YSFTIELR** | 1028.16 | **514.9** | **631.4** | **y_5_** | **75** | **23** |
|  |  |  |  | 514.9 | 778.5 | y_6_ | 75 | 21 |
| SIS Angpt-1 | **-** | **DAPHVEPDFSSQ(K*)** | 1464.46 | **489.1** | **457.3** | **y_4_** | **45** | **18** |
|  |  |  |  | 489.1 | 639.9 | y_11_^2+^ | 45 | 16 |
|  |  | LEIQLLENSLSTY(K*) | 1658.81 | 830.1 | 243.2 | b_2_ | 80 | 42 |
|  |  |  |  | 830.1 | 1062.7 | y_9_ | 80 | 33 |
|  |  | GHTGTAG(K*) | 735.71 | 368.9 | 195.1 | b_2_ | 85 | 24 |
|  |  |  |  | 368.9 | 441.2 | y_5_ | 85 | 20 |
| SIS sTie-2 | **-** | YIGGNLFTSAFT(R*) | 1456.53 | 729.1 | 1180.6 | y_11_ | 60 | 33 |
|  |  |  |  | 729.1 | 839.5 | y_7_ | 60 | 33 |
|  |  | **EEDAVIY(K*)** | 973.99 | **487.9** | **431.4** | **y_3_** | **75** | **18** |
|  |  |  |  | 487.9 | 318.1 | y_2_ | 75 | 21 |
|  |  | FSVAIFTVN(R*) | 1163.26 | 582.5 | 646.3 | y_5_ | 80 | 23 |
|  |  |  |  | 582.5 | 830.6 | y_7_ | 80 | 25 |
| SIS ANXA5 | **-** | **TPEELSAI(K*)** | 995.05 | **498.5** | **797.4** | **y_9_** | **70** | **23** |
|  |  |  |  | 498.5 | 447.8 | y_8_^2+^ | 70 | 25 |
|  |  | GAGTDDHTLI(R*) | 1165.15 | 389.4 | 519.3 | y_9_^2+^ | 45 | 14 |
|  |  |  |  | 389.4 | 512.4 | y_4_ | 45 | 23 |
|  |  | FITIFGT(R*) | 964.05 | 482.9 | 704.5 | y_6_ | 65 | 21 |
|  |  |  |  | 482.9 | 490.3 | y_4_ | 65 | 21 |
| SIS sP-sel | **-** | **GITSLPAPAV(R*)** | 1091.20 | **546.5** | **620.3** | **y_6_** | **45** | **21** |
|  |  |  |  | 546.5 | 472.3 | b_5_ | 45 | 16 |
|  |  | SNSAPG(K*) | 667.63 | 334.8 | 309.3 | y_3_ | 45 | 18 |
|  |  |  |  | 334.8 | 467.3 | y_5_ | 45 | 14 |
| SIS sTM | **-** | EVVLQHV**(R*)** | 989.06 | 330.8 | 421.2 | y_3_ | 60 | 19 |
|  |  |  |  | 330.8 | 324.7 | z_8_^3+^ | 60 | 8 |
|  |  | **LQGHLMTV(R*)** | 1064.20 | **355.7** | **467.5** | **z_8_^2+^** | **60** | **16** |
|  |  |  |  | 355.7 | 516.4 | y_4_ | 60 | 21 |
| SIS MAG | **-** | TQVVHESFQG**(R*)** | 1297.31 | 433.4 | 230.1 | b_2_ | 80 | 19 |
|  |  |  |  | 433.4 | 534.8 | y_9_^2+^ | 80 | 17 |
|  |  | **LLGDLGL(R*)** | 865.95 | **434.0** | **199.3** | **a_2_** | **60** | **22** |
|  |  |  |  | 434.0 | 271.1 | b_2_ | 60 | 17 |
|  |  | EFVYSE**(R*)** | 938.91 | 470.4 | 564.3 | y_4_ | 40 | 20 |
|  |  |  |  | 470.4 | 249.3 | a_2_ | 40 | 28 |
| SIS THBS-1 | **-** | SITLFVQED(R*) | 1217.26 | 609.4 | 803.5 | y_6_ | 75 | 26 |
|  |  |  |  | 609.4 | 201.2 | b_2_ | 75 | 23 |
|  |  | LVPNPDQ(K*) | 917.97 | 459.8 | 353.7 | y_6_^2+^ | 50 | 16 |
|  |  |  |  | 459.8 | 495.3 | y_4_ | 50 | 25 |
|  |  | **AQGYSGLSV(K*)** | 1017.06 | **509.5** | **598.3** | **y_6_** | **65** | **22** |
|  |  |  |  | 509.5 | 818.4 | y_8_ | 65 | 21 |
| SIS TAFI | **-** | YGFLLPE(R*) | 1004.07 | 502.9 | 411.3 | y_3_ | 80 | 21 |
|  |  |  |  | 502.9 | 524.4 | y_4_ | 80 | 21 |
|  |  | IYIGSSFE(K*) | 1051.11 | 526.5 | 775.4 | y_7_ | 75 | 21 |
|  |  |  |  | 526.5 | 277.2 | b_2_ | 75 | 22 |
|  |  | **YSFTIEL(R*)** | 1038.09 | **519.9** | **641.5** | **y_5_** | **75** | **23** |
|  |  |  |  | 519.9 | 788.4 | y_6_ | 75 | 21 |

^a^-At least two transitions for each peptide were monitored. The most intensive transitions of each selected peptide in plasma were added to the table. Transitions used for the quantification were shown in bold.
