# Supplementary material for: Distinct patterns of endothelial response to endotoxin in aged mice as compared to young mice
Source: GeroScience. 2025 Nov 26;48(2):1981–99. doi: 10.1007/s11357-025-01838-9 (PMC12972439; doi:10.1007/s11357-025-01838-9)
Supplement: Supplementary file 16 — (DOCX 41.7 KB) [file 11357_2025_1838_MOESM10_ESM.docx]

**Suppl. Table 3.1** Calibration curves, determination coefficients and linear ranges for all selected amino acid sequences specific for Angpt-1, sTie-2, ANXA5, sP-sel, sTM, MAG, THBS-1 and TAFI spiked into artificial plasma (N=4)

| **Protein** | **Target quantitative peptide sequence** | **Calibration curve** | **Determination coefficient (R^2^)** | **Linear range**  **(pmol/mL)** | **LLOQ**  **(pmol/mL)** |
| --- | --- | --- | --- | --- | --- |
| Angpt-1 | DAPHVEPDFSSQK | y=(0.0135 ± 0.0007)x+(0.0403 ± 0.0467) | 0.9986 | 0.3 - 200 | 0.3 |
| sTie-2 | EEDAVIYK | y=(0.0053 ± 0.0004)x+(0.0108 ± 0.0084) | 0.9985 | 0.3 - 200 | 0.3 |
| ANXA5 | TPEELSAIK | y=(0.0398 ± 0.0078)x+(0.0879 ± 0.0392) | 0.9994 | 0.3 - 200 | 0.3 |
| sP-sel | GITSLPAPAVR | y=(0.0082 ± 0.0005)x+(0.0276 ± 0.0025) | 0.9998 | 0.1 - 200 | 0.1 |
| sTM | LQGHLMTVR | y=(0.0065 ± 0.0010)x+(0.0394 ± 0.0326) | 0.9995 | 0.3 - 200 | 0.3 |
| MAG | LLGDLGLR | y=(0.0066 ± 0.0006)x+(0.9980 ± 0.1288) | 0.9990 | 0.3 - 200 | 0.3 |
| THBS-1 | AQGYSGLSVK | y=(0.2408 ± 0.0060)x+(0.0115 ± 0.0057) | 0.9992 | 2 - 4000 | 2 |
| TAFI | YSFTIELR | y=(0.2025 ± 0.0048)x+(0.0020 ± 0.0006) | 0.9999 | 2 - 4000 | 2 |

N- number of calibration curve replicates per each protein.
