# Supplementary material for: Distinct patterns of endothelial response to endotoxin in aged mice as compared to young mice
Source: GeroScience. 2025 Nov 26;48(2):1981–99. doi: 10.1007/s11357-025-01838-9 (PMC12972439; doi:10.1007/s11357-025-01838-9)
Supplement: Supplementary file 17 — (DOCX 55.4 KB) [file 11357_2025_1838_MOESM11_ESM.docx]

**Suppl. Table 4.1** Intra- and inter-day precision, accuracy and recovery for all selected amino acid sequences used in the quantitative analysis of Angpt-1, sTie-2, ANXA5, sP-sel, sTM, MAG and THBS-1 when spiked into artificial plasma

| **Protein** | **Target quantitative peptide sequence** | **Nominal conc.**  **(pmol/mL)** | | **Intra-day** | | | | **Inter-day** | | | | | **N** | **Recovery**  **(%)** | **Mean ± SD**  **(%)** |
| --- | --- | --- | --- | --- | --- | --- | --- | --- | --- | --- | --- | --- | --- | --- | --- |
|  |  |  |  | **N** | **Mean, measured conc.^A^ ± SD**  **(pmol/mL)** | **Accuracy (%)^a^** | **Precision (%)^b^** | | **N** | **Mean, measured conc.^B^ ± SD**  **(pmol/mL)** | **Accuracy (%)^a^** | **Precision (%)^b^** |  |  |  |
| Angpt-1 | DAPHVEPDFSSQK | 0.2 | 4 | | < LOQ | < LOQ | < LOQ | 9 | | < LOQ | < LOQ | < LOQ | 5 | < LOD | 106 ± 11 |
|  |  | 2 | 4 | | 2.0 ± 0.3 | 100.0 | 15.0 | 9 | | 2.0 ± 0.3 | 100.0 | 15.0 | 5 | 117.8 |  |
|  |  | 20 | 4 | | 21.0 ± 2.3 | 105.0 | 11.0 | 9 | | 20.9 ± 1.9 | 104.5 | 9.1 | 5 | 105.1 |  |
|  |  | 150 | 4 | | 150 ± 10 | 100.0 | 6.7 | 9 | | 154 ± 11 | 102.7 | 7.1 | 5 | 95.1 |  |
| sTie-2 | EEDAVIYK | 0.2 | 4 | | < LOQ | < LOQ | < LOQ | 9 | | < LOQ | < LOQ | < LOQ | 5 | <LOD | 97 ± 12 |
|  |  | 2 | 4 | | 2.2 ± 0.1 | 110.0 | 4.5 | 9 | | 2.0 ± 0.3 | 100.0 | 15.0 | 5 | 109.8 |  |
|  |  | 20 | 4 | | 20.3 ± 0.7 | 101.5 | 3.4 | 9 | | 20.5 ± 1.0 | 102.5 | 4.9 | 5 | 94.8 |  |
|  |  | 150 | 4 | | 152 ± 6 | 101.3 | 3.9 | 9 | | 151 ± 5 | 100.7 | 3.3 | 5 | 85.9 |  |
| ANXA5 | TPEELSAIK | 0.2 | 4 | | < LOQ | < LOQ | < LOQ | 9 | | < LOQ | < LOQ | < LOQ | 5 | < LOD | 106 ± 6 |
|  |  | 2 | 4 | | 2.0 ± 0.2 | 100.0 | 10.0 | 9 | | 2.0 ± 0.3 | 100.0 | 15.0 | 5 | 100.1 |  |
|  |  | 20 | 4 | | 18.5 ± 0.7 | 92.5 | 3.8 | 9 | | 21.1 ± 3.8 | 105.5 | 18.0 | 5 | 111.3 |  |
|  |  | 150 | 4 | | 150 ± 10 | 100.0 | 6.7 | 9 | | 154 ± 9 | 102.3 | 5.9 | 5 | 105.2 |  |
| sP-sel | GITSLPAPAVR | 0.2 | 4 | | 0.2 ± 0.03 | 100.0 | 15.0 | 9 | | 0.2 ± 0.03 | 108.6 | 15.0 | 5 | 121.4 | 111 ± 7 |
|  |  | 2 | 4 | | 1.9 ± 0.1 | 95.0 | 5.3 | 9 | | 1.9 ± 0.1 | 95.0 | 5.3 | 5 | 108.2 |  |
|  |  | 20 | 4 | | 19.4 ± 0.6 | 97.0 | 3.1 | 9 | | 19.5 ± 0.7 | 97.5 | 3.6 | 5 | 106.5 |  |
|  |  | 150 | 4 | | 150 ± 1 | 100.0 | 0.7 | 9 | | 149 ± 3 | 99.3 | 2.0 | 5 | 107.1 |  |
| sTM | LQGHLMTVR | 0.2 | 4 | | < LOQ | < LOQ | < LOQ | 9 | | < LOQ | < LOQ | < LOQ | 5 | <LOD | 108 ± 5 |
|  |  | 2 | 4 | | 1.8 ± 0.2 | 90.0 | 11.1 | 9 | | 1.8 ± 0.3 | 90.0 | 16.7 | 5 | 113.7 |  |
|  |  | 20 | 4 | | 20.9 ± 2.5 | 104.5 | 12.0 | 9 | | 19.6 ± 2.7 | 98.0 | 13.8 | 5 | 104.9 |  |
|  |  | 150 | 4 | | 147 ± 18 | 98.0 | 12.2 | 9 | | 152 ± 16 | 101.3 | 10.5 | 5 | 106.1 |  |
| MAG | LLGDLGLR | 0.2 | 4 | | < LOQ | < LOQ | < LOQ | 9 | | < LOQ | < LOQ | < LOQ | 5 | <LOD | 109 ± 13 |
|  |  | 2 | 4 | | 2.3 ± 0.1 | 115.0 | 4.3 | 9 | | 2.3 ± 0.2 | 115.0 | 8.7 | 5 | 118.8 |  |
|  |  | 20 | 4 | | 18.8 ± 2.5 | 94.0 | 13.3 | 9 | | 20.8 ± 3.8 | 104.0 | 18.3 | 5 | 93.4 |  |
|  |  | 150 | 4 | | 148 ± 8 | 98.7 | 5.4 | 9 | | 150 ± 6 | 100.0 | 4.0 | 5 | 113.7 |  |
| THBS-1 | AQGYSGLSVK | 3 | 4 | | 2.8 ± 0.3 | 93.3 | 10.7 | 9 | | 3.1 ± 0.4 | 103.3 | 12.9 | 5 | 97.9 | 91 ± 6 |
|  |  | 40 | 4 | | 42.4 ± 4.9 | 106.0 | 11.6 | 9 | | 42.2 ± 5.7 | 105.5 | 13.5 | 5 | 92.9 |  |
|  |  | 400 | 4 | | 368 ± 26 | 91.9 | 7.1 | 9 | | 389 ± 30 | 97.3 | 7.7 | 5 | 85.4 |  |
|  |  | 2000 | 4 | | 2069 ± 84 | 103.5 | 4.1 | 9 | | 2108 ± 122 | 105.4 | 5.8 | 5 | 87.3 |  |
| TAFI | YSFTIELR | 3 | 4 | | 3.3 ± 0.3 | 110.0 | 9.1 | 9 | | 3.3 ± 0.3 | 110.0 | 9.1 | 5 | 121.2 | 103 ± 14 |
|  |  | 40 | 4 | | 39.3 ± 0.4 | 98.3 | 1.0 | 9 | | 39.6 ± 1.2 | 99.0 | 3.0 | 5 | 97.0 |  |
|  |  | 400 | 4 | | 385 ± 8 | 96.3 | 2.1 | 9 | | 375 ± 15 | 93.8 | 4.0 | 5 | 106.5 |  |
|  |  | 2000 | 4 | | 2007 ± 79 | 100.4 | 3.9 | 9 | | 1985 ± 68 | 99.3 | 3.4 | 5 | 87.8 |  |

**^a^** RE (relative error; %) = 100 + {[(measured value – theoretical value)/theoretical value] × 100}.

**^b^** RSD (relative standard deviation; %) = SD/average × 100.

**^A^** Mean of 4 replicates at each concentration.

**^B^** Mean of 9 replicates for three precision and accuracy batches.

**N**- The number of repetitions.

**< LOQ**- detected values below Limit of Quantification

**< LOD**- detected values below Limit of Detection
