# Supplementary material for: Distinct patterns of endothelial response to endotoxin in aged mice as compared to young mice
Source: GeroScience. 2025 Nov 26;48(2):1981–99. doi: 10.1007/s11357-025-01838-9 (PMC12972439; doi:10.1007/s11357-025-01838-9)
Supplement: Supplementary file 18 — (DOCX 47.7 KB) [file 11357_2025_1838_MOESM12_ESM.docx]

**Suppl. Table 5.1** Normalized matrix effect (normalized ME; N=5), short**-**term and long**-**term stability calculated for all selected amino acid sequences used in the quantitative analysis of Angpt-1, sTie-2, ANXA5, sP-sel, sTM, MAG, THBS-1 and TAFI when spiked into artificial plasma

(N=3)

| **Protein** | **Target quantitative peptide sequence** | **Nominal conc.**  **(pmol/mL)** | **Normalized ME (%)** | **Short-term stability** | | | **Long-term stability** |
| --- | --- | --- | --- | --- | --- | --- | --- |
|  |  |  |  | **24 hours (%)** | **24 hours in autosampler (%)** | **3 cycles of freezing−thawing (%)** | **3 months (%)** |
| Angpt-1 | DAPHVEPDFSSQK | 0.2 | - | - | - | - | - |
|  |  | 2 | 90 | 108 | 87 | 90 | 116 |
|  |  | 20 | 95 | 103 | 104 | 108 | 96 |
|  |  | 150 | 88 | 93 | 94 | 93 | 101 |
| sTie-2 | EEDAVIYK | 0.2 | - | - | - | - | - |
|  |  | 2 | 98 | 100 | 109 | 114 | 92 |
|  |  | 20 | 89 | 100 | 96 | 101 | 100 |
|  |  | 150 | 98 | 101 | 100 | 97 | 105 |
| ANXA5 | TPEELSAIK | 0.2 | - | - | - | - | - |
|  |  | 2 | 101 | 96 | 91 | 89 | 88 |
|  |  | 20 | 92 | 97 | 103 | 103 | 110 |
|  |  | 150 | 89 | 92 | 106 | 105 | 114 |
| sP-sel | GITSLPAPAVR | 0.2 | 94 | - | - | - | - |
|  |  | 2 | 116 | 99 | 98 | 100 | 86 |
|  |  | 20 | 96 | 103 | 98 | 104 | 97 |
|  |  | 150 | 96 | 103 | 101 | 101 | 97 |
| sTM | LQGHLMTVR | 0.2 | - | - | - | - | - |
|  |  | 2 | 108 | 113 | 87 | 83 | 100 |
|  |  | 20 | 89 | 95 | 94 | 96 | 100 |
|  |  | 150 | 85 | 106 | 104 | 105 | 105 |
| MAG | LLGDLGLR | 0.2 | - | - | - | - | - |
|  |  | 2 | 85 | 109 | 102 | 99 | 92 |
|  |  | 20 | 117 | 97 | 96 | 103 | 108 |
|  |  | 150 | 101 | 101 | 97 | 101 | 99 |
| THBS-1 | AQGYSGLSVK | 3 | 89 | - | - | - | - |
|  |  | 40 | 105 | 95 | 91 | 90 | 111 |
|  |  | 400 | 110 | 103 | 97 | 100 | 110 |
|  |  | 2000 | 95 | 103 | 105 | 105 | 106 |
| TAFI | YSFTIELR | 3 | 99 | - | - | - | - |
|  |  | 40 | 106 | 97 | 108 | 103 | 103 |
|  |  | 400 | 88 | 98 | 101 | 101 | 112 |
|  |  | 2000 | 102 | 102 | 99 | 104 | 90 |

N- number of replicates at each concentration.
