# Supplementary material for: Distinct patterns of endothelial response to endotoxin in aged mice as compared to young mice
Source: GeroScience. 2025 Nov 26;48(2):1981–99. doi: 10.1007/s11357-025-01838-9 (PMC12972439; doi:10.1007/s11357-025-01838-9)
Supplement: Supplementary file 19 — (DOCX 74.1 KB) [file 11357_2025_1838_MOESM13_ESM.docx]

**Distinct patterns of endothelial response to endotoxin in aged mice as compared to young mice**

***Short title: Aging alters the pattern of endothelial response in endotoxemia in mice***

Joanna Suraj-Prażmowska^a*^, Magdalena Sternak^a^, Anna Kurpińska^a^, Anna Bar^a^, Izabela Czyżyńska-Cichoń^a^, Kelly Ascencao^d^, Ewa Niedzielska-Andres^c^, Elżbieta Buczek^a^, Łukasz Mateuszuk^a^, Agnieszka Karaś^a^, Maria Walczak^c^, Csaba Szabo^d^, Stefan Chlopicki^a,b*^

*^a^ Jagiellonian University, Jagiellonian Centre for Experimental Therapeutics (JCET),*

*Krakow, Poland*

*^b^ Jagiellonian University, Jagiellonian University Medical College, Faculty of Medicine, Chair of Pharmacology, Krakow, Poland*

*^c^ Jagiellonian University Medical College, Faculty of Pharmacy, Chair and Department of Toxicology, Krakow, Poland*

*^d^ University of Fribourg, Faculty of Science and Medicine, Department of Oncology, Microbiology and Immunology, Section of Pharmacology, Fribourg, Switzerland*

*Corresponding authors: Jagiellonian Centre for Experimental Therapeutics (JCET), Jagiellonian University, Bobrzynskiego 14, 30-348, Krakow, Poland Tel.: +48 12 664 54 81; Fax: +48 12 297 46 15 E-mail address: joanna.suraj@jcet.eu, [stefan.chlopicki@jcet.eu](mailto:stefan.chlopicki@jcet.eu)

**SUPPLEMENTAL MATERIAL**

**Chromatographic and mass spectrometric conditions of the microLC/MS-MRM method used for the quantitative determination of plasma levels of the panel of 20 protein/peptide biomarkers reflecting various aspects of endothelial function**

In this study, extension of the microLC/MS-MRM method was established based on a UPLC coupled on-line to a mass spectrometer via a positive electrospray ionization (ESI) source with the aim to add additional 8 biomarker proteins to the previously described panel of 12 biomarkers detecting endothelial dysfunction [1,2].

The best separation of all 20 analytes (12 previously validated biomarkers and 8 newly validated biomarkers) was obtained using an ACE, C8 analytical column (1 × 150 mm, 5 µm, 300Å, Advanced Chromatography Technologies Ltd, Aberdeen, Scotland) at 30°C of column oven temperature. The mobile phases consisted of 0.1% FA in ACN (A) and 0.1% FA in H_2_O (v/v) (B) and were delivered in a gradient elution at the flow rate of 100 µL/min. Gradient elution was as follows: 98% B from 0 to 5 min, 98% – 20% B from 5 to 25 min, hold 20% B from 25 to 30 min, 20% – 98% B from 30 to 32 min and 18 min for column equilibration at 98% B. The total time of analysis was 50 minutes. The sample injection volume was modified compared to the last version of the method and was set to 5 µL [1,2]. The operating parameters for the mass spectrometer used in the MRM method were as follows: ion spray voltage: 5500 V, source temperature: 400°C, curtain gas: 25 psi, ion source gas 1: 35 psi, ion source gas 2: 50 psi. The specified precursor peptide-to-fragment ion transitions and product ions selected for the quantification of proteins as well as the values for collision energy (CE) and declustering potential (DP) are listed in Suppl. Table 2.1.

**Solutions**

***Preparation of peptide mixture naturally occurring within the selected eight proteins***

Stock solutions of analytes were prepared as follows: 1 mg of each lyophilized sample was dissolved in 1 mL of 20% solution of acetonitrile (ACN) in deionized water to give a concentration of 1 mg/mL (concentrations of stock solutions for selected analytes expressed in µmol/mL are presented in Suppl. Table 1.1). Final concentrations of obtained working stock solutions were 25 nmol/mL for Angpt-1, sTie-2, ANXA5, sP-sel, sTM, MAG and 50 nmol/mL for THBS-1 and TAFI.

Working standard solutions of the analytes added to calibration curve (CC) samples were 0.25, 0.5, 1.5, 2, 20, 50, 200, 500 and 1000 pmol/mL for Angpt-1, sTie-2, ANXA5, sP-sel, sTM and MAG and 5, 10, 30, 40, 400, 1000, 3400, 5000 and 20000 pmol/mL for THBS-1 and TAFI. Final concentrations of prepared CC samples were five times lower than their corresponding concentrations for working standard solutions of analytes. The CC samples were made at nine concentrations: 0.05, 0.1, 0.3, 0.4, 4, 10, 40, 100 and 200 pmol/mL for Angpt-1, sTie-2, ANXA5, sP-sel, sTM and MAG and at nine concentrations: 1, 2, 6, 8, 80, 200, 680, 1000 and 4000 pmol/mL for THBS-1 and TAFI.

The concentrations of working standard solutions for quality control (QC) samples were 1, 10, 100 and 750 pmol/mL for Angpt-1, sTie-2, ANXA5, sP-sel, sTM and MAG and 15, 200, 2000 and 10000 pmol/mL for THBS-1 and TAFI. Final concentrations of prepared QC samples were also five times lower than their concentrations of working standard solutions for QC samples and were made at four concentrations: 0.2, 2, 20 and 150 pmol/mL for Angpt-1, sTie-2, ANXA5, sP-sel, sTM and MAG and at four concentrations: 3, 40, 400 and 2000 pmol/mL for THBS-1 and TAFI.

***Preparation of SIS mixture***

The stock solutions of stable isotope-labelled internal standards were prepared by dissolving 1 mg of specified SIS in 20% solution of ACN in deionized water (1 mg/mL; concentrations of stock solutions for selected SIS expressed in µmol/mL are presented in Suppl. Table 1.1).

The concentrations of SIS working stock solutions were individually selected for all analyzed SIS taking into account the endogenous concentration of eight chosen analytes measured in plasma. For Angpt-1, sTie-2, ANXA5, sP-sel, sTM and MAG were 169.90 pmol/mL, 205.34 pmol/mL, 50.25 pmol/mL, 229.11 pmol/mL, 126.38 pmol/mL, 133.13 pmol/mL, for THBS-1 and TAFI were 3932.91 pmol/mL and 5975.68 pmol/mL, respectively. Final concentrations of SIS working solutions in CC samples and studied samples were five times lower than in SIS working stock solutions. For Angpt-1, sTie-2, ANXA5, sP-sel, sTM and MAG were 33.98 pmol/mL, 41.07 pmol/mL, 10.05 pmol/mL, 45.82 pmol/mL, 25.28 pmol/mL, 26.63 pmol/mL and for THBS-1 and TAFI were 786.58 pmol/mL and 1195.14 pmol/mL, respectively.

An aqueous solution of 25 mM ammonium bicarbonate (NH_4_HCO_3_) was prepared according to the protocol described in the work of Suraj et al. [1]. In the experiment, the aqueous solution of 25 mM NH_4_HCO_3_ was also used for the preparation of working solutions of 100 mM DTT, 100 mM IAM, 50 mM TCEP and 10% sodium deoxycholate. For enzymatic digestion, SGM trypsin at the concentration of 400 µg/mL prepared in 25 mM NH_4_HCO_3_ was applied.

Artificial plasma used for the preparation of CC and QC samples was made according to the European Standard PN-EN ISO 10993-15:2019 [3]. The pH of the artificial plasma was between 7.35 and 7.45. To estimate the impact of plasma proteins at physiological concentrations on the digestion efficiency, bovine serum albumin (BSA) at the concentration of 30 mg/mL was also added [4,5].

**Method validation**

The method validation was carried out for eight selected proteins such as Angpt-1, sTie-2, ANXA5, sP-sel, sTM, THBS-1 and TAFI. The finally chosen validation parameters: linearity range, intra- and inter-day precision and accuracy, recovery of analytes, matrix effect and stability of analytes were calculated as an average of at least three repetitions, instead of the five recommended by the European Medicines Agency (EMA) and the Food and Drug Administration (FDA) guidelines but with the required acceptance criteria [6,7]. Detailed information about the obtained results from method validation was presented below.

***Linearity***

The standard calibration curves were obtained by the analysis of nine CC samples for Angpt-1, sTie-2, ANXA5, sP-sel, sTM and MAG and eight CC samples for THBS-1 and TAFI prepared in artificial plasma and plotting the peak area ratio of specific amino acid sequences for native proteins and exogenous SIS versus the corresponding nominal analyte concentrations. The used concentrations of selected peptides are shown in Supplementary Information. Calibration curve samples were freshly prepared for each of five analytical runs. As required, the accepted criteria of each standard concentration was ± 15% deviation from the nominal one, except for the lower limit of quantification (LLOQ), where 20% is acceptable.

***Recovery, precision and accuracy***

The recoveries of selected analytes were evaluated at four concentration levels by analysis of five separately prepared sample sets. For Angpt-1, sTie-2, ANXA5, sP-sel, sTM and MAG the values of QC samples estimated in one sample set were 0.2, 2, 20 and 150 pmol/mL. Additionally, the values of analyzed QC samples in one sample set for THBS-1 and TAFI were 3, 40, 400 and 2000 pmol/mL. To calculate the recovery, the peak area ratios of the analyte/SIS in artificial plasma samples spiked with standard solutions prior to the micro solid phase extraction (µSPE) step with those spiked after the µSPE procedure were compared.

The inter- and intra-day precision and accuracy were estimated for selected peptide sequences at four different QC levels- 0.2, 2, 20 and 150 pmol/mL for Angpt-1, sTie-2, ANXA5, sP-sel, sTM and MAG and 3, 40, 400 and 2000 pmol/mL for THBS-1 and TAFI. For the intra-day precision and accuracy analyses, three replicates of QC samples at each concentration were performed. Additionally, the inter-day precision and accuracy were assessed totally for nine analytical runs analyzed with three consecutive days (each day three analytical runs). The criteria for acceptability of the data included accuracy within 85–115% from the nominal values and precision within ± 15% relative standard deviation (RSD).

***Matrix effect***

The normalized matrix effect (normalized ME) was evaluated using QC samples prepared in artificial plasma. Normalized ME was separately calculated for eight selected proteins in four concentrations. Each concentration was prepared in 5 replicates. For Angpt-1, sTie-2, ANXA5, sP-sel, sTM and MAG the used QC levels were 0.2, 2, 20 and 150 pmol/mL while for THBS-1 and TAFI were 3, 40, 400 and 2000 pmol/mL, respectively. This parameter was calculated by dividing the ratio between the analyte peak area obtained for samples spiked with standard solutions after matrix µSPE extraction and the analyte peak area registered for pure standard solutions and the ratio between the SIS peak area obtained for samples spiked with standard solutions after matrix µSPE extraction and the SIS peak area registered for pure standards.

***Stability***

The analyte short-term stability (24 hours), autosampler stability and three freeze-thaw cycles’ stability were examined using artificial plasma samples spiked with standard solutions to obtain the final concentration of the analytes of 2, 20 and 150 pmol/mL for Angpt-1, sTie-2, ANXA5, sP-sel, sTM and MAG and 40, 400 and 2000 pmol/mL for THBS-1 and TAFI. The first set of prepared samples was immediately analyzed. The second set of samples was frozen at -20°C and thawed after 24 hours. The autosampler stability was assessed by analyzing selected QC samples left in the autosampler for 24 h at 4°C. Finally, freeze-thaw stability was verified through three cycles of freezing-thawing (-20°C to room temperature as one cycle). To calculate the stability of the analytes, the peak area ratio of analyte/SIS obtained for thawed samples was compared to the peak area ratio of analyte/SIS estimated for freshly prepared samples. The stability of all analytes was assessed based on the three measurements per concentration. The long-term stability was determined by analyzing selected QC samples left at -20°C for three months. The criteria for acceptability of the data includes the accuracy within 85–115% of the nominal values, and the precision within ± 15% of the RSD.

**Results of validation process**

***Linearity***

The 8 validated proteins included to refreshed method showed good linearity over the concentration range of 0.1–200 pmol/mL for sP-sel and 0.3–200 pmol/mL for Angpt-1, sTie-2, ANXA5, sTM and MAG. Additionally, for THBS-1 and TAFI, satisfactory linearity was observed in the range of 2–4000 pmol/mL. The best fit of standard curves for all analytes with excellent determination coefficients (R^2^ > 0.9985) was obtained applying a 1/x weighting algorithm. Regression equations and the lowest concentrations accepted as LLOQ for each analyte are presented in Suppl. Table 3.1. The deviation of the LLOQ was within ± 20% of the nominal concentration and ± 15% for standards other than LLOQ, which was in line with EMA and FDA requirements.

***Recovery, precision and accuracy***

The intra- and inter-day precision expressed as RSD did not exceed for most proteins ± 20% for LLOQ and ± 15% for other QC concentrations. For ANXA5 and MAG the calculated value of RSD for the concentration 20 pmol/mL was 18.0% and 18.3%, respectively. Moreover, the intra- and inter-day method accuracy was 80–120% for LLOQ and 85–115% for higher concentrations of QC samples. The estimated values for method accuracy and precision are summarized in Suppl. Table 4.1.

The mean recoveries were 106 ± 11%, 97 ± 12%, 106 ± 6%, 111 ± 7%, 108 ± 5%, 109 ± 13%, 91 ± 6% and 103 ± 14% for Angpt-1, sTie-2, ANXA5, sP-sel, sTM, MAG, THBS-1 and TAFI, respectively.

What was important, in case of Angpt-1, sTie-2, ANXA5, sTM and MAG to calculate the recovery, precision and accuracy, only three values of QC samples were used. The reason was the linearity range for Angpt-1, sTie-2, ANXA5, sTM and MAG, where the value of first curve point was higher than the value of the first prepared QC sample (0.2 pmol/mL).

***Matrix effect***

The normalized ME was separately calculated for all analytes. Developed results for individual compounds are presented in Suppl. Table 5.1. The lowest normalized ME calculated for Angpt-1, sTie-2, ANXA5, sTM and MAG at low quality control (LQC) =  2 pmol/mL was observed for MAG (85%), whereas at high quality control (HQC) = 150 pmol/mL was noted for sTM (85%). Additionally, LQC (0.2 pmol/mL) and HQC (150 pmol/mL) for sP-sel was 94% and 96%, respectively. Interestingly, normalized ME was slightly higher than expected for sP-sel at a concentration of 2 pmol/mL and for MAG at a concentration of 20 pmol/mL and was 116% and 117%, respectively.

The lowest normalized ME calculated for THBS-1 and TAFI at low quality control (LQC) = 3 pmol/mL was observed for THBS-1 (89%), whereas at high quality control (HQC) = 2000 pmol/mL was noted for TAFI (102%).

***Stability***

The stability of Angpt-1, sTie-2, ANXA5, sP-sel, sTM and MAG after 24 h storage in the freezer (−20 °C) measured for three concentrations (2 pmol/mL, 20 pmol/mL and 150 pmol/mL) was within the range of 92–113%. For THBS-1 and TAFI the stability investigated for 40 pmol/mL, 400 pmol/mL and 2000 pmol/mL was within the range of 95–103%. Additionally, almost all analytes prepared in artificial plasma were stable after 24 h of processing in the autosampler batch and after three cycles of freezing-thawing. Only the stability of sTM at a concentration of 2 pmol/mL after three cycles of freezing-thawing was slightly lower than required and was 83%. The long-term stability of the analytes after three months of storage in −20 °C was also carefully determined. The criteria for acceptability were highly fulfilled for each analyzed compound (Suppl. Table 5.1).

Stability changes in the concentration of different QC samples for analytes were very comparable for the values obtained for both short- and long-term storage. In stability studies, the chemical nature of peptides and proteins should be considered. During storage in different types of containers, they can bind to each other and to the materials from which the containers are made. Therefore, to prevent analyte loss, fresh standard solutions were prepared prior to each analysis.

**References to Supplementary Information**

[1] Suraj J, Kurpińska A, Olkowicz M, Niedzielska-Andres E, Smolik M, Zakrzewska A, et al. Development, validation and application of a micro–liquid chromatography–tandem mass spectrometry based method for simultaneous quantification of selected protein biomarkers of endothelial dysfunction in murine plasma. J Pharm Biomed Anal 2018;149:465–74. https://doi.org/10.1016/j.jpba.2017.11.023.

[2] Suraj J, Kurpińska A, Sternak M, Smolik M, Niedzielska-Andres E, Zakrzewska A, et al. Quantitative measurement of selected protein biomarkers of endothelial dysfunction in plasma by micro-liquid chromatography-tandem mass spectrometry based on stable isotope dilution method. Talanta 2019;194:1005–16. https://doi.org/10.1016/J.TALANTA.2018.10.067.

[3] ISO 10993-15:2019(en), Biological evaluation of medical devices — Part 15: Identification and quantification of degradation products from metals and alloys. Online Browsing Platform (OBP) 2019. https://www.iso.org/obp/ui/#iso:std:iso:10993:-15:ed-2:v1:en (accessed May 7, 2025).

[4] Fernández I, Peña A, Del Teso N, Pérez V, Rodríguez-Cuesta J. Clinical Biochemistry Parameters in C57BL/6J Mice after Blood Collection from the Submandibular Vein and Retroorbital Plexus. J Am Assoc Lab Anim Sci 2010;49:202.

[5] Schnell MA, Hardy C, Hawley M, Propert KJ, Wilson JM. Effect of blood collection technique in mice on clinical pathology parameters. Hum Gene Ther 2002;13:155–61. https://doi.org/10.1089/10430340152712700.

[6] Bioanalytical Method Validation Guidance for Industry | FDA. Center for Drug Evaluation and Research Center for Veterinary Medicine 2018:1–41. https://www.fda.gov/regulatory-information/search-fda-guidance-documents/bioanalytical-method-validation-guidance-industry (accessed May 7, 2025).

[7] Bioanalytical method validation - Scientific guideline | European Medicines Agency (EMA). European Medicines Agency 2009:1–23. https://www.ema.europa.eu/en/bioanalytical-method-validation-scientific-guideline (accessed December 19, 2024).
